# Amyloid fibril structures of tau: Conformational plasticity of the second microtubule-binding repeat

**DOI:** 10.1126/sciadv.adh4731

**Published:** 2023-07-14

**Authors:** Nadia El Mammeri, Pu Duan, Aurelio J. Dregni, Mei Hong

**Affiliations:** Department of Chemistry, Massachusetts Institute of Technology, 170 Albany Street, Cambridge, MA 02139, USA.

## Abstract

The intrinsically disordered protein tau associates with microtubules in neurons but aggregates into cross–β amyloid fibrils that propagate in neurodegenerative brains. Different tauopathies have different structures for the rigid fibril core. To understand the molecular basis of tau assembly into different polymorphs, here we use solid-state nuclear magnetic resonance (NMR) spectroscopy to determine the structure of a tau protein that includes all microtubule-binding repeats and a proline-rich domain. This P2R tau assembles into well-ordered filaments when induced by heparin. Two- and three-dimensional NMR spectra indicate that R2 and R3 repeats constitute the rigid β-sheet core of the fibril. Unexpectedly, the amino-terminal half of R2 forms a β-arch at ambient temperature (24°C) but a continuous β-strand at 12°C, which dimerizes with the R2 of another protofilament. This temperature-dependent structure indicates that R2 is conformationally more plastic than the R3 domain. The distinct conformational stabilities of different microtubule-binding repeats give insight into the energy landscape of tau fibril formation.

## Teaser

Solid-state NMR data reveal that tau's R2 repeat has high conformational plasticity during amyloid fibril formation.

## INTRODUCTION

**Fig. 1.**
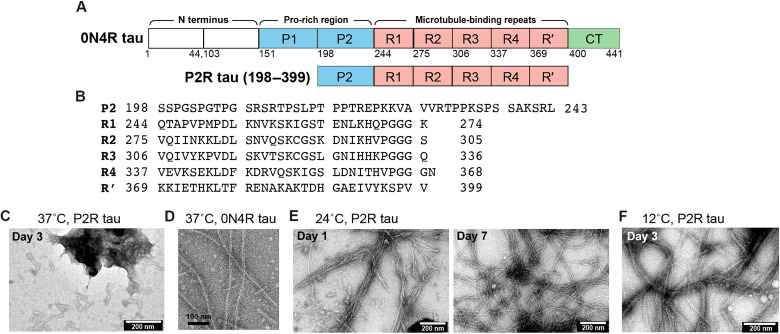
Amino acid domains of tau and the morphology of P2R tau fibrils. (**A**) Sequence diagram of 0N4R tau and P2R tau. (**B**) Amino acid sequence of P2R tau. (**C** to **F**) Negative-stain TEM images of heparin-induced tau aggregates. (C) P2R tau forms amorphous aggregates at 37°C under shaking at 300 rpm. (D) Full-length 0N4R tau forms long filaments at 37°C under shaking at 1400 rpm. (E) P2R tau forms long filaments at 24°C under rocking at 50 rpm. The filaments formed within 1 day and became longer and more homogeneous after 7 days. (F) P2R tau forms long and bundled filaments at 12°C under shaking at 55 rpm.

Tau is an intrinsically disordered protein that stabilizes microtubules (MTs) to promote axonal transport (*1*). In Alzheimer’s disease (AD), this highly soluble protein aggregates into intracellular neurofibrillary tangles, whose spreading in the brain is the basis of current neuropathological staging of AD. Tau amyloid formation also occurs in other neurodegenerative diseases such as corticobasal degeneration (CBD) and progressive supranuclear palsy (PSP) (*2*). Native tau is highly charged and soluble. The protein contains a negatively charged N-terminal domain (NT), followed by a positively charged proline-rich region (PRR), a positively charged MT-binding region, and a short negatively charged C-terminal domain (CT) (Fig. 1). The MT-binding region contains either three (R1, R3, and R4) or four repeats (R1, R2, R3, and R4) of 31 to 32 residues each, followed by a pseudo-repeat R′. The NT contains zero, one, or two 29-residue inserts (0N, 1N, or 2N). These sequence variations together give rise to six tau isoforms in human brains, among which an abundant isoform, 0N4R tau (*3*), has a net charge of +15 at pH 7. How this charged and intrinsically disordered protein aggregates into β-sheet amyloid fibrils in diseased brains is not yet understood.

Although the precise molecular mechanism of tau aggregation is still largely unknown, many structures of aggregated tau have now been determined. Early transmission electron microscopy (TEM) images of paired helical filaments (PHFs) and straight filaments of AD tau showed a fuzzy exterior, which can be removed by pronase to reveal an ordered core (*4*–*7*). High-resolution cryo–electron microscopy (cryo-EM) data (*8*) indicate a C-shaped molecular structure for this ordered AD tau fibril core, which spans 73 residues in the R3, R4, and a portion of the R′ domains. Eight β-strands in this C-shaped structure form parallel-in-register hydrogen bonds across hundreds to thousands of molecules, giving the spine of the filaments. This rigid core accounts for only ~20% of full-length 0N4R and 0N3R tau, whereas the rest of the protein is too disordered to be resolved in the cryo-EM density maps. If the rest of the protein is fully present in the fuzzy coat of these PHF tau filaments, then this fuzzy coat would be negatively charged except for the PRR, and the R1 and R2 repeats. However, whether the rest of the protein is present in the untreated PHF tau filaments is uncertain. Recent proteomic and other biochemical data show that the progression of AD is correlated with increasing truncation of the NT and CT (*9*, *10*). The CT is cleaved at intermediate stages of the progression of AD, whereas the NT is cleaved at late stages. This suggests that cleavage of negatively charged domains of tau might accelerate the nucleation and subsequent prion-like propagation of the β-sheet core. Similar to AD, other tauopathies also exhibit small fibril cores, which contain 73 to 107 amino acid residues within the MT-binding repeats (*11*–*16*).

Recombinant tau fibrils formed in vitro provide good opportunities for investigating how truncations and posttranslational modifications might nucleate and propagate tau aggregates in brains. Tau fibrils can be induced in vitro using anionic cofactors such as the sulfated glycosaminoglycan heparin (*7*, *17*). To understand how cleavage of the NT and CT affects the aggregation process, we need to first characterize the structure of full-length tau fibrils. Recently, we conducted a systematic study of the structure and dynamics of full-length 0N4R and 0N3R tau using solid-state nuclear magnetic resonance (ssNMR) spectroscopy (*16*, *18*, *19*). We found that heparin-induced 0N4R tau and 0N3R tau fibrils are non-twisting and are monomorphic as long as the protein is pure and intact during fibril growth (*18*). When proteolysis occurs during fibril growth to cause truncation products, then polymorphic fibrils form (*20*). The ssNMR spectra of these full-length tau fibrils show a single set of chemical shifts for the rigid residues, indicating a single molecular conformation for the fibril core. The 0N4R tau fibril core consists of a β-arch for the R2 and R3 domains (V275–S324), whereas the 0N3R tau has a four-layered β-sheet core that spans R3, R4, R′, and the entire CT (T263–L441) (*16*). Therefore, both full-length tau isoforms exclude the NT from the rigid core. This NT exclusion is also seen in all brain tau fibrils known to date (*15*). The CT is similarly excluded from all ex vivo tau fibril cores but is included in the rigid core of heparin-induced 0N3R tau fibrils. These findings suggest that the NT and CT may have inhibitory effects on tau aggregation. In contrast, the R′ domain, which is C-terminal to the R1-R4 repeats, is partly included in all ex vivo tau fibril cores known so far. R′ is also the center of the MT-immobilized domain of tau (*21*), implicating this domain to be crucial in the transition of tau from the intrinsically disordered and functional state to the well-ordered and dysfunctional state (*22*). N-terminal to the regular repeats, the cationic PRR contains many Ser-Pro and Thr-Pro residue pairs that are phosphorylated in AD PHF tau and is important for stabilizing tau association with MTs (*23*, *24*).

These structural and biophysical data suggest that a tau construct that includes all four MT-binding repeats, the pseudo-repeat, and part of the PRR may be useful for studying the aggregation propensity of the MT-binding regions of tau. Here, we investigate such a construct, termed P2R tau (residues 198 to 399), which spans the P2, R1, R2, R3, R4, and R′ domains. Using ssNMR, we have characterized the conformation, dynamics, and three-dimensional (3D) fold of heparin-induced P2R tau fibrils. Unexpectedly, we find that the protein adopts two distinct but related rigid-core conformations at temperatures that are only ~12°C apart. This result indicates an exquisite sensitivity of the conformation of the R2 repeat to environmental conditions, giving insight into the conformational variability of this domain in brain-extracted 4R tau fibrils.

## RESULTS

### Heparin-induced P2R tau fibrils are monomorphic but temperature dependent

P2R tau is a 202-residue protein spanning P2, R1, R2, R3, R4, and R′ domains of full-length tau (Fig. 1, A and B). We overexpressed the protein in *Escherichia coli* and purified it by heat denaturation, affinity column chromatography, and high-performance liquid chromatography (HPLC) (fig. S1). Fibrils were assembled by mixing tau monomers (0.1 mg/ml) with heparin (0.125 mg/ml) under three conditions: at 37°C with shaking at 300 rpm, at 24°C with rocking at 50 rpm, and at 12°C with shaking at 55 rpm. At 37°C, only amorphous fibrils of less than 50 nm length are observed (Fig. 1C). This behavior differs qualitatively from full-length 0N4R tau, which forms long and ordered filaments under the same temperature and with even faster shaking (Fig. 1D) (*18*). Reducing the temperature to 24°C allowed P2R tau to develop long filaments with a 9 nm width (Fig. 1E). Further reducing the temperature to 12°C slowed down fibril growth and yielded long filaments that are bundled (Fig. 1F). These observations indicate that the removal of the NT and CT increases the aggregation propensity of tau, which, in turn, requires lower temperatures and slower kinetics to form ordered β-sheet assemblies.

### Ambient-temperature and low-temperature P2R tau fibrils have distinct β sheet cores

**Fig. 2.**
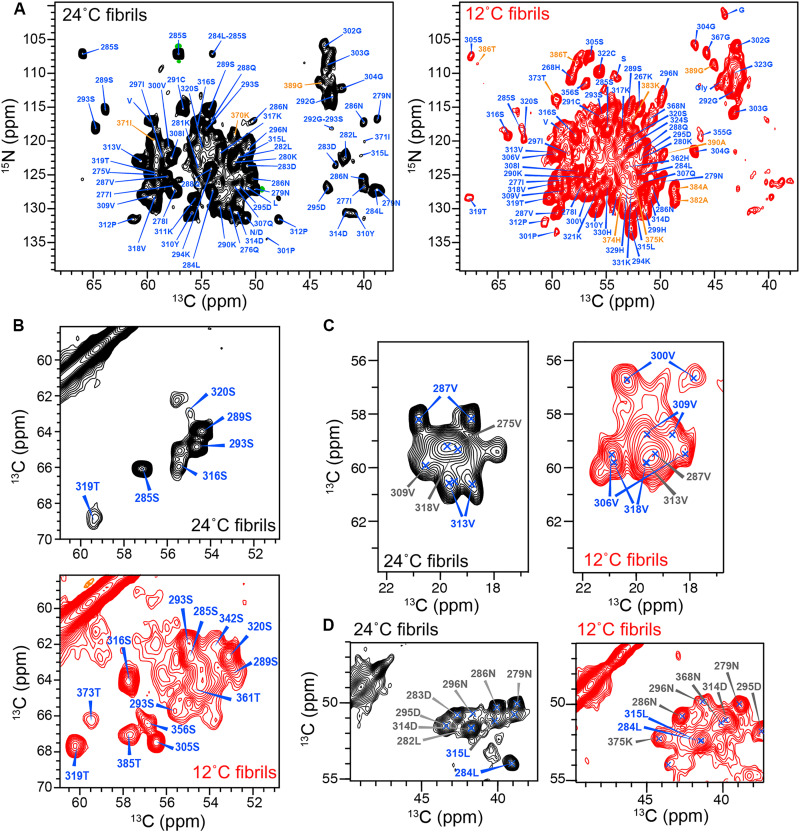
Comparison of the 2D CC and NC correlation spectra of ambient-temperature and low-temperature P2R tau fibrils, produced at 24° and 12°C, respectively. (**A**) 2D NC spectra. Orange assignments denote R′ residues. (**B**) Ser/Thr region of the 2D CC spectra. The 12°C fibril shows a larger number of peaks than the 24°C fibril in both the 2D CC and NC spectra, indicating that fibrilization at lower temperature leads to a larger β-sheet core. (**C**) Val Cα-Cγ region of the 2D CC spectra. Some residues show two resolved methyl Cγ chemical shifts (assigned in blue), indicating that these side chains are locked into tight contact with other residues, unable to undergo rotameric jumps. (**D**) Leu Cα-Cβ region of the 2D CC spectra. L284 and L315 peaks (assigned in blue) show β-sheet chemical shifts in the 12°C fibril, while L284 displays coil-like chemical shifts in the 24°C fibril. Resonance assignments are obtained from 3D correlation spectra.

To characterize the structures of the rigid cores of these P2R tau fibrils, we measured ssNMR spectra. 1D ^13^C cross-polarization (CP) spectra selectively detect immobilized residues, whereas direct-polarization (DP) spectra give approximately quantitative intensities of both mobile and immobile residues. The 12°C fibril shows higher CP intensities than DP intensities compared to the 24°C fibril (fig. S1D), indicating that low-temperature fibril growth produced a larger rigid core. 2D ^15^N─^13^C (NC) correlation spectra exhibit about 50 peaks for the 24°C fibril and about 65 peaks for the 12°C fibril (Fig. 2A), and the 2D ^13^C─^13^C (CC) correlation spectra (Fig. 2B) show 7 Ser and Thr peaks for the 24°C fibril but at least 15 peaks for the 12°C fibril. These observations suggest that the low-temperature fibrils contain a larger rigid core compared to the ambient temperature fibrils. Both samples display narrow ^13^C and ^15^N linewidths, which are 0.5 to 0.8 parts per million (ppm) for ^13^C and 0.8 to 1.2 ppm for ^15^N, implying that both fibrils have well-ordered molecular conformations.

**Fig. 3.**
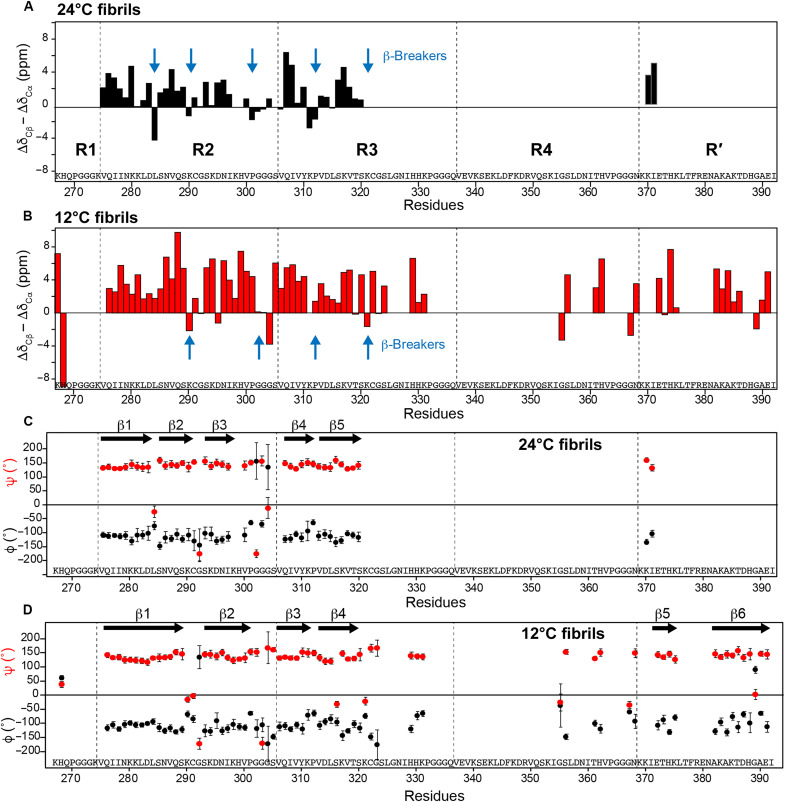
Secondary structures of P2R tau fibrils depend on temperatures. (**A**) Cβ and Cα secondary chemical shift differences of the 24°C P2R fibril. Positive differences are indicative of a β-strand conformation. Five β-strands can be identified between Lys^274^ and Ser^320^. Breaks between β-strands are indicated by blue arrows. These β-strand locations in the amino acid sequence are similar to those of the full-length 0N4R tau. (**B**) Cβ and Cα secondary chemical shift differences of the 12°C P2R fibril. β-strands are found not only in the R2 and R3 repeats but also in R4 and R′ repeats.

We assigned the ^13^C and ^15^N chemical shifts sequence-specifically using 3D ^15^N─^13^C correlation experiments, including NCACX, NCOCX, and CONCA (fig. S2). For the 24°C fibril, we assigned 48 residues, most of which reside between K274 and S320 (table S1) in the R2 and the first half of R3 (Fig. 3, A and C). In addition, we observed signals for ^369^KKI^371^ (Fig. 2A), indicating that a small part of R′ is immobilized. For the 12°C fibril, we assigned 72 residues, which span R2, R3, and part of the R′ domain (Fig. 2, B and D, and table S2). Most of the strong correlation peaks in the spectra are sequentially assigned, and a single set of chemical shifts is observed for all residues except for G304 and S293 in the 12°C fibril (Fig. 2A and fig. S3). These results indicate that P2R tau fibrils predominantly adopt a single molecular conformation at both temperatures. In the 24°C fibril, C291 signals are observed, but C322 signals are absent, while both Cys residues are assigned in the 12˚C fibril (table S1). All observed Cys chemical shifts indicate the reduced state (*25*), ruling out the existence of disulfide bonds in these samples. This is consistent with the observation of only reduced Cys residues in brain-extracted tau fibrils. Five spin systems in each fibril remain unassigned due to insufficient sensitivity or resonance overlap.

In addition to the size difference of the rigid cores, the two P2R tau fibrils exhibit distinct Val side-chain chemical shifts: Some Val residues have two resolved Cγ chemical shifts, whereas other Val residues have a single Cγ chemical shift. The former is indicative of the Val side-chain being locked into close contact with another residue, whereas the latter suggests rotameric averaging of the two methyl groups. The high-temperature fibrils exhibit a single Cγ chemical shift for V309 and two Cγ chemical shifts for V287 (Fig. 2C), whereas the 12°C fibril has the opposite behavior. These data suggest that the V287 side chain is conformationally constrained in the 24°C fibril, whereas the V309 side chain is constrained in the 12°C fibril. We also detected interesting differences in the Leu chemical shifts. L284 and L315 are matching residues in the R2 and R3 repeats. L315 exhibits β-sheet chemical shifts in both the ambient and low-temperature fibrils; in comparison, L284 displays random-coil chemical shifts in the 24°C fibril but β-sheet chemical shifts in the 12°C fibril (Fig. 2D). Together, these findings indicate that the fibrillization temperature affects not only the size but also the detailed structural arrangement of the rigid core. Comparing the two P2R tau fibrils with full-length 0N4R and 0N3R tau (figs. S3 and S4), we found that the R3 hexapeptide residues ^306^VQIVYK^311^ display similar chemical shifts, indicating that this segment is the invariant structural core among these tau constructs.

The assigned Cα and Cβ chemical shifts (fig. S5A) allow us to obtain the secondary structures of P2R tau fibrils (Fig. 3 and tables S3 to S5) (*26*). The secondary chemical shift difference between Cβ and Cα is positive for β-sheet conformations and negative for α-helical conformations. We identified five β-strands in the 24°C fibril. The first three strands are separated by L284 and the ^291^CGS^293^ triplet in the R2 repeat. The R2 ^300^VPGGG^304^ segment separates the β3 strand from the β4 strand at the R3 hexapeptide. Residue P312 marks a break before the β5 strand (^314^DLSKVTS^320^), which ends at the second ^322^CGS^324^ motif. In the 12°C P2R tau fibrils, the chemical shifts delimit four β-strands in the R2 and R3 domains and indicate additional β-strands in the R4 and R′ repeats (Fig. 3B). The ^291^CGS^293^ triplet and the ^300^VPGGG^304^ segment form similar β-sheet breakers, but L284 exhibits canonical β-sheet chemical shifts, making the N-terminal half of R2 a long continuous β-strand, in contrast to the 24°C fibril. Chemical shift comparisons between the two fibrils (fig. S5B) indicate that the R2 domain exhibits larger chemical shift differences than the R3 domain.

### Long-range contacts reveal the 3D structure of P2R tau fibrils

**Fig. 4.**
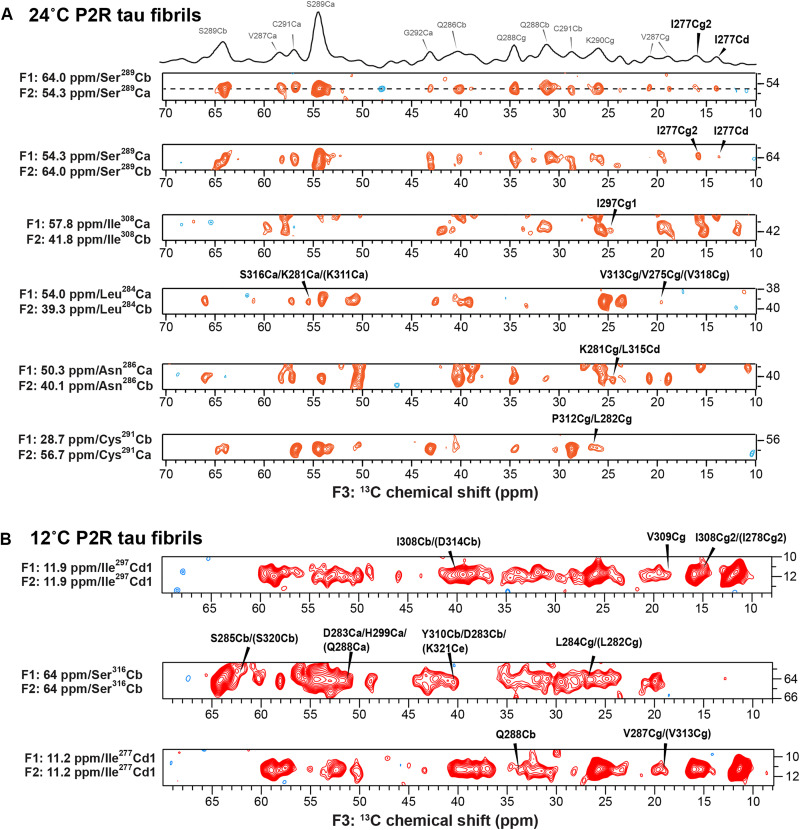
Representative strips from the 3D CCC spectra of P2R tau fibrils. (**A**) The 24°C P2R tau fibril. The 1D cross section of the top strip is shown as an example. Long-range contacts are assigned in black and bolded. Intra-residue and sequential cross peaks are assigned in gray for the top strip. (**B**) The 12°C P2R tau fibril. Only long-range contacts are assigned. The 3D experiment was conducted using DREAM and CORD for polarization transfer during the two mixing times for the 24°C fibril sample but with CORD during both mixing times for the 12°C fibril sample.

To determine the tertiary structures of the high- and low-temperature P2R tau fibrils, we measured long-range inter-residue contacts using 3D CCC experiments with a mixing time of 400 ms. For the 24°C fibril, we identified 36 long-range contacts (table S6) and 103 medium-range contacts, in addition to about 500 sequential and intra-residue correlations. We observed correlations between the β1 and β2 strands (I277–S289), the β2 and β4 strands (S289–P312), and the β3 and β4 strands (I297–V308) (Fig. 4A). These contacts indicate a three-layered topology for the fibril core: β2 and β3 strands lie in the middle layer, flanked by β4-β5 strands on one side and β1 on the other. For the 12°C fibril, the 3D CCC spectrum (Fig. 4B) yielded 19 long-range correlations (table S7), among which four indicate close contact between I297 in the middle of R2 and V309 in the R3 hexapeptide. About 200 sequential and intra-residue correlations were also observed. We detected correlations between I277 or I278 in the R2 hexapeptide and V287 or Q288 in the middle of R2. Because these contacts occur within a continuous β1 strand, they can only be satisfied if two protofilaments pack together, with the β1 strand lining the interface of an antiparallel dimer.

**Fig. 5.**
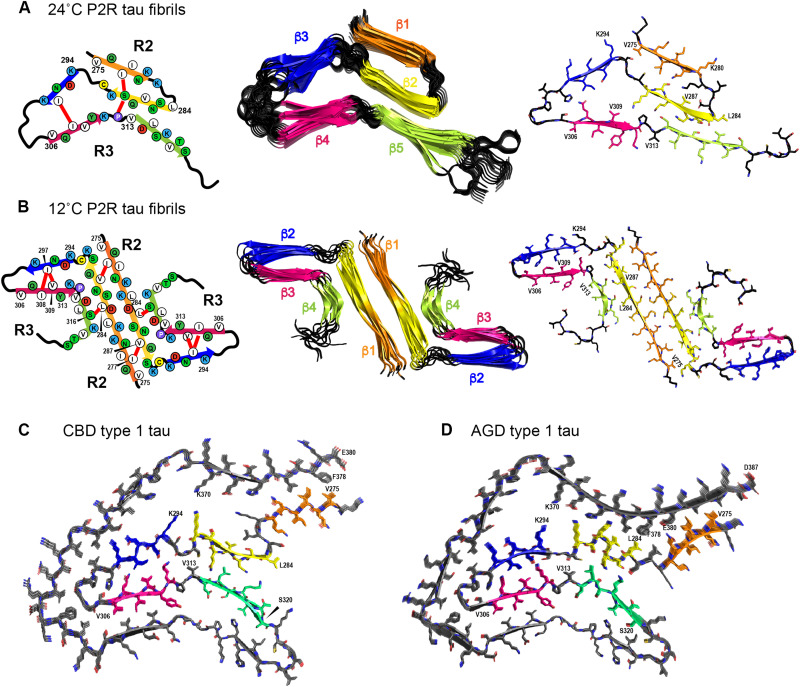
Structural models of heparin-fibrillized P2R tau fibril cores compared to brain-derived tau fibril cores. All structures are shown with the ^300^VPGGGS^305^ segment (black) on the left, followed by the R3 hexapeptide motif ^306^VQIVYK^311^ (magenta), and with residues 291 to 320 oriented similarly to facilitate comparison. (**A**) Structural model the 24°C P2R tau fibril. Left: Schematic model. Unambiguous long-range correlations are indicated by red lines. Middle: Ten lowest-energy structures. Breaks between β-strands are shown in black. Right: Lowest-energy structural model. The first residue of each β strand is indicated. (**B**) Structural model of the 12°C P2R tau fibril. Left: Schematic model. Middle: Ten lowest-energy structures. Right: Lowest-energy structural model. (**C**) Structure of ex vivo CBD type 1 tau fibril core (*14*). (**D**) Structure of ex vivo AGD type 1 tau fibril core (*15*).

Combining all these long-range correlations and chemical-shift derived ϕ and ψ torsion angles (table S3 and S4), we calculated the structures of both high- and low-temperature P2R tau fibrils using XPLOR-NIH (table S8). For the 12°C fibril, we assumed a 2_1_-screw symmetric dimer, based on the fact that most brain-derived dimeric tau aggregates exhibit this symmetry (*8*, *14*, *15*). The lowest-energy structural ensemble of the 24°C fibril (Table 1) shows a three-layered β-sheet core spanning residues K274–S320, in which two of the three layers are formed by the R2 domain via a sharp turn at ^281^KLDL^284^ (Fig. 5A). The ^301^PGGG^304^ motif at the end of R2 forms a second turn that directs the R3 domain to pack against R2 as the third layer of the rigid core. In comparison, the lowest-energy ensemble of the 12°C fibril (Fig. 5B) shows a four-layered β sheet core constructed by two protofilaments, whose interface is lined by the long β1 strand from V275 to S289. The β1 strand then turns sharply at the ^291^CGS^293^ triplet to form a well-ordered β-arch between the β2 strand and the R3 hexapeptide. P312 is the turning point for the next β strand, which stacks against the long β1 strand.

**Table 1.** NMR and refinement statistics for ambient-temperature and low-temperature P2R tau fibrils.

	24°C fibril	12 °C fibril
**NMR distance and dihedral constraints**		
Distance constraints		
Total NOE	228 × 5	118 × 10
Intra-residue	0	0
Inter-residue	144 × 5	26 × 10
Medium range (2 ≤ |*i* − *j*| ≤ 4)	103 × 5	0
Unambiguous long-range (|*i* − *j*| ≥ 5)	3 × 5	4 × 10
Ambiguous long-range (|*i* − *j*| ≥ 5)	33 × 5	12 × 10
Intermolecular	0	3 × 10
Total dihedral angle restraints		
ϕ	42 × 5	49 × 10
ψ	42 × 5	49 × 10
**Structure statistics**		
Violations (means ± SD)		
Distance constraints (Å)	0.06 ± 0.03	0.3 ± 0.18
Dihedral angle constraints (°)	1.05 ± 0.26	0.72 ± 0.14
Max. dihedral angle violation (°)	6.3	5.1
Max. distance constraint violation (Å)	1.6	1.5
Deviations from idealized geometry		
Bond lengths (Å)	0.005 ± 0.0003	0.005 ± 0.0004
Bond angles (°)	0.57 ± 0.018	0.51 ± 0.013
Impropers (°)	0.37 ± 0.03	0.29 ± 0.01
Average pairwise RMS deviation (Å)*		
Heavy	2.84	3.43
Backbone	2.47	3.17

*Pairwise root mean square deviation (RMSD) was calculated as the lowest RMSD between the most representative structure (medoid model) and the other 9 structures among the 10 lowest-energy structures. For the 24°C fibril, structure 2 was used to compare with the others. For the 12°C fibril, structure 4 was used to compare with the others.

### Water accessibility of the P2R tau fibrils reveals the fibrils topology

**Fig. 6.**
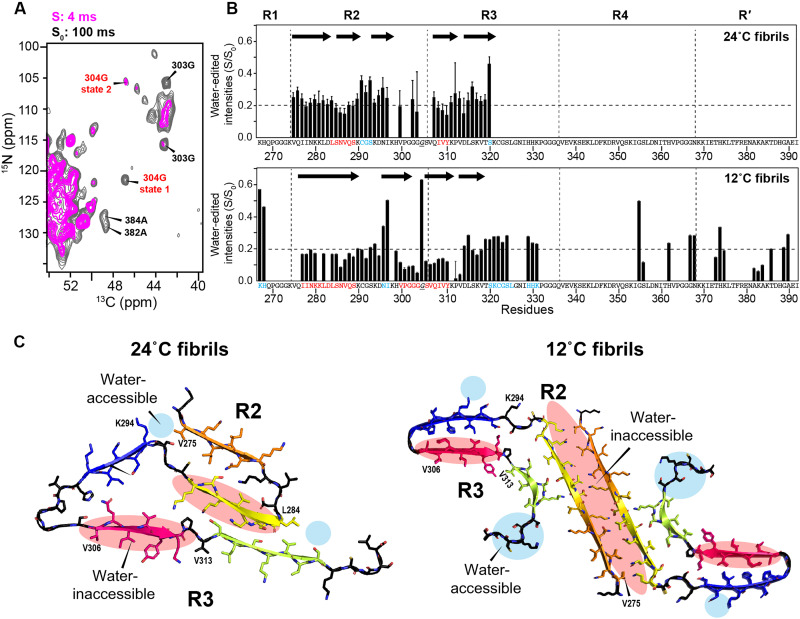
Water accessibilities of P2R tau fibrils differ for the two P2R tau fibrils. (**A**) Representative region of the water-edited 2D NC spectra of the 12°C fibril, measured with ^1^H mixing times of 4 ms (magenta) and 100 ms (gray). Water-accessible residues preferentially retain their intensities in the 4-ms spectrum. (**B**) Intensity ratios (S/S_0_) between the 4-ms (S) and 100-ms (S_0_) water-transferred spectra of the 24°C (top) and 12°C (bottom) fibrils. Dashed lines at S/S_0_ = 0.2 guide the eye for comparing the hydration of the two samples. The N-terminal half of R2 in the 12°C fibril shows particularly low water accessibilities. (**C**) Lowest-energy structures of the 24° and 12°C P2R tau fibrils, showing the most water-accessible regions (blue ovals) and the most water-inaccessible regions (red ovals). The N-terminal half of R2 in the 12°C fibril has uniformly low hydration, consistent with this segment forming the interface of two protofilaments.

Water-edited NMR experiments provide additional restraints to the structures of the two P2R tau fibrils. We measured the water accessibilities by transferring the water ^1^H magnetization to protein amide protons and detecting the result in 2D ^15^N─^13^C correlation spectra (table S9). The relative intensities between the 4-ms and 100-ms water-transferred spectra represent a site-resolved hydration map of the fibril core (Fig. 6A and fig. S6). For the 24°C fibril, the ^291^CGS^293^ turns between β2 and β3 strands, and the C-terminal S320 displays the highest water accessibilities (Fig. 6B), whereas ^285^SNV^287^ in the β2 strand and ^308^IVYK^311^ in the β4 strand are the least water-accessible residues. The former is consistent with the sequestration of β2 by β1 and β5 strands on the two sides, whereas the latter suggests that the R3 hexapeptide motif is sequestered by additional residues, potentially ^369^KKIE^372^ in R′ (Fig. 6C). For the 12°C fibril, we found uniformly low water accessibilities for the first 15 residues of R2, supporting the dimer structure of the 12°C fibril. This dehydrated segment is followed by two well-hydrated residues, N296 and I297, before turning into a dehydrated ^300^VPGGG^304^ segment. Thus, the junction between the β3 and β4 strands is protected from water in the 12°C fibril. This dehydration is consistent with the sequestration of ^300^VPGGG^304^ by an outer layer in ex vivo CBD and argyrophilic grain disease (AGD) fibrils (Fig. 5, C and D). G304 exhibits two N-Cα correlation peaks: The major peak (state 1) has an unusually large ^15^N chemical shift of 121.6 ppm (fig. S5C) and is water inaccessible, indicating that this unusual conformation is surrounded by other residues. In comparison, the minor peak (state 2) has a regular ^15^N chemical shift and is water accessible, suggesting that a small fraction of fibrils contain a disordered and water-exposed ^300^VPGGG^304^ loop.

## DISCUSSION

### Thermodynamic and kinetic implications of the temperature-dependent P2R tau structures

The P2R fibril structures obtained here give insight into the effects of the fuzzy coat on tau aggregation and provide evidence for different conformational stabilities of different MT-binding repeats. When the NT and CT are removed, tau forms amorphous, short, and clumped aggregates at 37°C (Fig. 1C). At the same temperature, under similar monomer and heparin concentrations, full-length 0N4R tau assembles into ~13-nm-wide and hundreds of nanometer-long filaments (*18*). This indicates that the removal of the NT and CT accelerates tau aggregation, such that nucleation of well-ordered hydrogen-bonded β-strands may be either entropically disfavored or heterogeneous. Reducing the temperatures from 37° to 24° and 12°C restored the assembly of β-sheet filaments but gave rise to two distinct but related fibril structures. The 24°C fibril predominantly consists of single filaments, whereas the 12°C fibril is laterally bundled. Chemical shifts and long-range correlation data indicate that the N-terminal half of R2 folds into a β-arch with a turn at L284 in the 24°C fibril, whereas the same segment folds into a continuous β-strand in the 12°C fibril (Fig. 3). The R2 β-arch in the high-temperature fibril is evidenced by long-range contacts between I277 and S289 and non–β sheet torsion angles at L284 (Fig. 3C). In the 12°C fibril, similar long-range contacts between I277 and V287/Q288 are also observed, but the continuous β-sheet conformation for this segment (Fig. 3D) indicates that these contacts are intermolecular in nature. We propose a model in which these inter-residue contacts are formed by two protofilaments that stack with 2_1_-screw symmetry. Although cryo-EM and scanning TEM can give information about the symmetry of amyloid assemblies, the extensive lateral bundling of the 12°C fibrils (Fig. 1F) prohibits their use. The conformationally plastic N-terminal half of R2 is capped by the ^291^CGS^293^ motif, after which the protein adopts a similar strand-turn-strand conformation at both temperatures (Fig. 4, A and B). We did not observe long-range contacts involving ^322^CGS^324^ and its C-terminal residues at either temperature, indicating that the protein is more disordered from this position to the C terminus. However, β strand chemical shifts are observed for C322 and many residues in R4 and R′ domains at 12°C, indicating that these segments are immobilized at this temperature. In comparison, few signals for residues C-terminal to ^322^CGS^324^ are detected in the 24°C fibril, indicating that the protein is more dynamic at the higher temperature.

These data indicate that different MT-binding repeats of tau have distinct conformational plasticity: The R3 repeat is the most stable amyloidogenic repeat, followed by R2. The R3 strand-turn-strand conformation is largely invariant in both P2R tau fibrils and full-length 0N4R tau (table S5 and fig. S7). In contrast, the N-terminal half of R2 is structurally plastic and can bend onto itself like a hairpin or straighten into a β strand that dimerizes. In full-length 0N4R tau, the same R2 segment shows intermediate disorder, with ^282^LD^283^ forming a loop whose signals are undetectable in the ssNMR spectra (*18*). The MT-binding repeats of tau are pseudo-repeats, and the R2 and R3 domains exhibit interesting amino acid sequence differences. The R3 hexapeptide ^306^VQIVYK^311^ contains a highly amyloidogenic Tyr^310^. This residue engages in steric zipper interactions with other side chains through π-π and CH-π stacking in most tau fibrils (*8*, *15*) and in crystals of this hexapeptide (*27*). The R2 hexapeptide ^275^VQIINK^280^ replaces this aromatic residue with an Asn, which may be less able to form side-chain interactions that stabilize β-sheet conformations. In addition, the charge distribution of the two repeats differs. The R2 repeat contains five Lys residues and two Asp residues. Among these, a ^280^KK^281^ doublet follows the R2 hexapeptide and is conformationally variable among different brain tau structures (fig. S8). This suggests that the ionic environment may influence the structure of this cationic Lys pair. The R3 domain contains four nonconsecutive Lys residues and one Asp. A ^311^KP^312^ pair follows the R3 hexapeptide and is the site of a kink in most tau fibril structures. These amino acid sequence differences may contribute to the distinct conformational stabilities of the R3 and R2 domains.

Although the R2 repeat is conformationally more plastic than R3, we observed a predominant set of chemical shifts at both temperatures, indicating that the two fibril structures do not coexist. Thus, there is a substantial energy barrier between the three-layered structure and the four-layered dimer structure. This high energy barrier is consistent with current understanding of the free energy landscapes of amyloid formation. Coarse-grained molecular dynamics simulations of Alzheimer's β amyloid (Aβ) fibrils found a small number of distinct minima that are separated by high energy barriers (*28*). These energy barriers can be understood in terms of the molecular events required for fibril elongation. Elongation of an existing fibril requires unstructured monomers to fold into a specific conformation, activation of the molecules on the fibril ends to a state competent for accepting new monomers (*29*), and binding of the newly folded monomer to the activated fibril end by establishing hydrogen bonds and side-chain interactions (*30*). The energies associated with these folding, activation, and binding events are substantial (*28*). Experimental measurements of fibril growth kinetics at different temperatures bear out this theoretical consideration. Temperature-dependent Aβ fibril elongation rates have been measured using quasi-elastic light scattering (*29*) and atomic force microscopy (*31*). These studies found a 20-fold acceleration from 4° to 24°C and fivefold acceleration from 24° to 37°C, indicating a large activation energy of ~95 kJ/mol.

Similar thermodynamic considerations may also underpin different nucleation rates of different fibril polymorphs. Amyloid fibril nucleation is complex (*32*), involving both primary and secondary nucleation mechanisms and both homogeneous and heterogeneous events. It has been shown for Aβ that secondary nucleation has a much weaker temperature dependence than primary nucleation, suggesting that the temperature sensitivity of the P2R tau fibril structure may result from differences in the primary nucleation kinetics between the three-layered structure and the four-layered polymorph. The intramolecular R2 β-arch structure seen at 24°C leaves multiple side chains such as Q276, I278, and K280 unconstrained by other side chains except for the next molecule in the hydrogen-bonded chain, whereas the four-layered dimer structure at 12°C engages all side chains in this domain in tight interactions. Therefore, we speculate that the formation of the three-layered nucleus may be entropically favored over the formation of the four-layered dimer nucleus, which leads to its faster nucleation at high temperature. Classical nucleation theory predicts the existence of maxima for nucleation, elongation, and overall crystal growth (*33*). This theory has found experimental support in the nucleation rates of glucagon fibrils, measured as the inverse of the lag time, which show maxima at intermediate temperatures (*34*). Correspondingly, glucagon fibrils exhibit temperature-dependent fibril polymorphs, like P2R tau. We hypothesize that the entropically favored three-layered β sheet core may have a higher temperature of maxima for nucleation and elongation compared to the four-layered dimer core and thus outgrows the four-layered dimeric fibril core at higher temperatures.

The P2R tau (residues 198 to 399) studied here is similar to K32 tau (198 to 400), originally designed to investigate tau interactions with MTs (*35*). Recently, a K32 tau assembly was studied using solution and ssNMR (*36*). The 2D CC spectrum of the sample shows much broader linewidths than that found here for P2R tau. As a result, comparisons with shorter tau constructs, including K19 and two chimeric tau constructs, were used to infer the rigid core conformation of K32. Those data were interpreted as the P2 domain being immobilized in K32 (*36*). Our data clearly rule out the participation of P2 in the rigid core of the fibrils obtained here. The K32 fibrils in the previous study were grown at 37°C from a 100 μM monomer solution. At the same temperature, with different buffer and shaking conditions, we found that P2R tau forms amorphous aggregates even at a 20-fold lower monomer concentration (4.7 μM). Ordered β-sheet filaments only formed at lower temperatures. The electron micrograph of the previous K32 sample showed amorphous features, and the characteristic β-sheet chemical shifts of Tyr^310^ that are common in tau amyloid fibrils (*16*, *18*) were absent from the 2D CC spectrum of K32 tau. These observations suggest that the partial immobilization of the P2 domain in the previous study may be specific to the assembly condition used to produce those amorphous K32 tau fibrils.

### Similarities and differences between P2R tau structures and brain 4R tau structures

The P2R tau fibril core structures show many similarities to 4R tau fibrils obtained from CBD, AGD, PSP, and globular glial tauopathy brains (Fig. 4, C and D, and fig. S8) (*15*). All four brain tau fibrils conserve the tight packing between the R3 hexapeptide and R2 residues ^294^KDNIK^298^, similar to the P2R tau structures seen here. The most variable segment in these brain 4R tau fibrils is the R2 hexapeptide ^275^VQIINK^280^, which stacks against R′ residues ^374^HKLTFR^379^ in CBD tau but shifts by six residues to stack against ^378^FRENAKA^384^ in AGD tau. The R2 ^281^KLDL^284^ segment is the variable hinge that controls the orientation and position of the R2 hexapeptide relative to the other β strands in the protein. This hinge position and R2 structural variability are in excellent agreement with the current P2R tau data. The main difference between CBD and AGD tau lies in the L284 conformation: L284 is part of a turn in the CBD structure but a β-strand residue that stacks against the R′ repeat in the AGD structure. This is similar to the temperature-dependent P2R structural models.

All four brain tau aggregates have a larger fibril core than P2R tau: They start from approximately residue 272 and end between residues 380 and 387 in R′. In P2R tau, we did not resolve long-range contacts involving residues C-terminal to S320 to define the structure of the R4 and R′ domains relative to the R3 and R2 domains. However, we detected rigid β-sheet R4 and R′ residues in the 12°C fibril. Moreover, water-edited spectra of the 12°C fibrils indicate that the R3 hexapeptide and the ^300^VPGGG^304^ turn are inaccessible to water (Fig. 6), implying that additional proteinaceous segments protect this domain from solvent exposure. We hypothesize that the immobilized but disordered residues in R4 and R′ may be responsible for shielding the ^300^VPGGG^304^ and the R3 hexapeptide from water. Future experiments at even lower temperatures may allow the identification of the positions of R4 and R′ repeats in the structure. These P2R tau data are consistent with the observation that the R3 domain is sequestered by an outer layer of R4 and R′ residues in all brain-derived 4R tau structures.

Our in vitro P2R tau structures, together with the ex vivo brain 4R tau structures, suggest that pure-isoform 4R tau folds into a small number of conformations. Understanding the intrinsic amino acid sequence basis and extrinsic environmental factors that drive the formation of these different structures is important for controlling and diverting fibril formation. Our data indicate that the removal of the disordered NT and CT led to in vitro fibril structures that more closely resemble the brain tau filaments. The temperature dependence of the P2R tau fibril structures reveals a conformational plasticity of the R2 domain, similar to the structural variability of R2 in brain 4R tau aggregates. The L284 region might thus be useful as a target of small-molecule inhibitors to disaggregate tau fibrils. Our data indicate that systematic analyses of precisely fibrillized in vitro tau give valuable insights into the energy landscape of tau amyloid fibril formation. The fact that the fuzzy coat–removed tau replicates the conformation of the innermost region of the brain tau aggregates also implies that tau truncation may be one of the mechanisms for the formation of pathological tau aggregates in human brains (*37*).

## MATERIALS AND METHODS

### Cloning, expression, and purification of P2R tau

The gene encoding P2R tau contains an N-terminal His_6_ tag, a thrombin cleavage site (10 residues), a tobacco etch virus (TEV) cleavage site (6 residues), and residues 198 to 399 of tau. Following TEV cleavage, a Gly residue remains before the N-terminal S198 in the final purified protein. This gene was cloned into a pET-28a vector and transfected into *E. coli* BL21(DE3) competent cells (New England Biolabs). A starter culture was grown in 50 ml of LB medium containing kanamycin (50 μg/ml). After overnight growth at 37°C with shaking at 220 rpm, the 50-ml overnight culture was used to inoculate 1 liter of LB medium containing kanamycin (50 μg/ml). Cells were grown at 37°C and 220 rpm until optical density at 600 nm reached 1.0, then the cells were spun down at 1000*g* and 4°C for 20 min, and the cell pellet was resuspended in 1 liter of minimal medium containing M9 salts, ^15^NH_4_Cl (1 g/liter), ^13^C-labeled glucose (2 g/liter), 1 mM MgSO_4_, 0.1 mM CaCl_2_, kanamycin (50 mg/ml), vitamin, and mineral supplements. Cells were grown at 37°C under shaking at 220 rpm for 2 hours, and, then, protein expression was induced with 1 mM isopropyl-β-d-thiogalactopyranoside (IPTG). At this point, another ^13^C-labeled glucose (1 g/liter) was added to the medium to give a total of ^13^C-labeled glucose (3 g/liter). Expression proceeded for 18 hours at 30°C under shaking at 180 rpm. Expression of 0N4R tau was induced with 0.5 mM IPTG and lasted 5 hours at 37°C with shaking at 180 rpm.

The His_6_-thrombin-TEV-P2R tau fusion protein was purified using Ni^2+^ affinity column chromatography. Then, the His6-thrombin-TEV tag was removed using TEV protease, and the native P2R tau was purified by reverse-phase HPLC. In detail, cells were spun down at 6000*g* and 4°C for 15 min. The pellet was resuspended in 40 ml of a lysis buffer containing 1× phosphate-buffered saline (PBS) (Sigma-Aldrich) [137 mM NaCl, 2.7 mM KCl, 8 mM Na_2_HPO_4_, and 2 mM KH_2_PO_4_ (pH 7.4)], 2 mM dithiothreitol (DTT), 0.05 mM phenylmethylsulfonyl fluoride (PMSF), and one tablet of cOmplete protease inhibitor cocktail (Roche). The cells were lysed by sonication on ice (5-s on, 5-s off for 10 min, 550 Sonic Dismembrator, Thermo Fisher Scientific). The lysate was boiled for 10 min and then spun at 20,000*g* for 60 min to remove cell debris and aggregates of heat-sensitive cellular proteins. The supernatant containing the fusion protein was loaded on 5 ml of Profinity IMAC resin that had been charged with Ni^2+^ (Bio-Rad, CA, USA) and washed with the binding buffer [1× PBS, 2 mM DTT, and 20 mM imidazole (pH 8)]. After loading, the resin was washed with 20 column volumes of binding buffer, and, then, the proteins were eluted from the column using 5 column volumes of the elution buffer [1× PBS, 2 mM DTT, and 400 mM imidazole (pH 8)]. The eluted fraction was loaded onto a desalting column (Econo-Pac 10 DG, Bio-Rad, CA, USA) to exchange buffer for the TEV cleavage buffer [50 mM tris, 150 mM NaCl, 2 mM DTT, 0.05 mM EDTA, and 0.05 mM PMSF (pH 8)]. The solution was then diluted to the fusion protein (~0.5 mg/ml) to prevent aggregation during cleavage. Subsequently, TEV protease was added at a ratio of 3:100 (w:w) with respect to the fusion protein, and the cleavage reaction proceeded for 16 hours at 4°C with gentle rocking. After cleavage, 2 M guanidinium chloride was added to the solution and then loaded onto 5 ml of Ni^2+^-charged resin that had been prewashed with the binding buffer. The flow-through containing the P2R tau protein was collected and then purified on a reverse-phase HPLC using a preparative C3 column (250-mm length, 21.2-mm inside diameter, 7-μm particle size, 300-Å pore size, Higgins analytical) and an acetonitrile gradient of 5 to 95% in 35 min. The eluted P2R tau fractions were >95% pure as determined by SDS–polyacrylamide gel electrophoresis with Coomassie blue staining. The fractions were pooled and lyophilized to give the P2R tau powder. The yield of the expression and purification was ~15 mg of protein per liter of M9 culture.

### Fibril formation and ssNMR sample preparation

The amount of P2R tau was measured gravimetrically with an estimated uncertainty of 15%. We used this method of quantification because P2R tau has a small extinction coefficient (2980 M^−1^ cm^−1^) due to its lack of Trp and the small number of other aromatic residues, which makes absorbance measurements unreliable. [U-^13^C, ^15^N]-labeled P2R tau powder was dissolved in 1× PBS buffer containing 2 mM DTT to reach a concentration of 2 mg/ml and then bath-sonicated for 5 min to remove small aggregates. This monomer solution was transferred to 50-ml Falcon tubes and diluted to 0.1 mg/ml (4.7-μM monomer), and, then, heparin was added to a final concentration of 0.125 mg/ml (Santa Cruz Biotechnology, sc203075; 8000 to 25,000 Da).

Three temperature and agitation conditions were used for fibril growth: (i) 37°C with shaking at 300 rpm (2.54-cm orbit); (ii) 24°C with rocking at 50 rpm; and (iii) 12°C with shaking at 55 rpm (2.54-cm orbit). In conditions (i) and (iii), we strapped 10 Falcon tubes (50-ml) each containing 25 ml of solution horizontally on an incubator platform (Excella E25, New Brunswick Excella) to induce a “sloshing” movement of the solution along the tube axis. In condition (2), the 10 Falcon tubes (50-ml) were strapped horizontally on a rocker platform (Corning LSE Platform Rocker, Double Platform) to induce a similar sloshing movement of the solution. For conditions (ii) and (iii), the rocking and shaking rates were chosen to cause similar amounts of agitation. For condition (i), the high temperature and fast shaking were designed to mimic the previously used conditions to produce full-length 0N4R tau (*18*) and 0N3R tau fibrils (*16*). To minimize variations in the parameters, we chose the same 2.54-cm orbit as for condition (iii) and used the maximum rate achievable by the incubator platform.

The solutions were incubated for 3 days but became cloudy after about 1 day. The samples prepared at 12° and 24°C were concentrated by ultracentrifugation at ~112,000*g* for 1 hour and 4°C using a fixed-angle rotor (Beckman TLA55). Hydrated pellets were spun into 3.2-mm Revolution NMR pencil rotors using a funnel made of pipette tips and a swinging-bucket centrifuge at 4000*g* for 5 min. The rotors contained ~36 mg of hydrated mass in which ~20 mg was estimated to be P2R tau fibrils.

### TEM of P2R tau fibrils

Unconcentrated P2R tau fibril solutions were adsorbed onto freshly glow-discharged, 200-mesh formvar/carbon-coated copper grids (Ted Pella), extensively washed with water to remove any trace of PBS buffer, and stained with 0.7% (w/v) uranyl formate for 15 s. TEM images were taken on an FEI Tecnai T12 electron microscope.

### ssNMR experiments and analysis

All ssNMR spectra were measured on a Bruker Avance 800-MHz (18.8-T) spectrometer at the Francis Bitter Magnet Laboratory using a Black Fox 3.2-mm HCN MAS probe. Sample temperatures were measured using the water ^1^H chemical shift, which was calibrated using DSS (sodium trimethylsilylpropanesulfonate) (*38*). ^13^C chemical shifts were referenced externally to the adamantane CH_2_ chemical shift at 38.48 ppm on the tetramethylsilane scale. ^15^N chemical shifts were referenced to the ^15^N peak of N-acetylvaline at 122.00 ppm on the liquid ammonia scale.

1D ^13^C MAS spectra were measured using different polarization transfer methods to detect residues with different mobilities. A ^1^H─^13^C CP spectrum measured with a contact time of 70 μs was used to detect the rigid residues, while a CP spectrum measured with a contact time of 800 μs was used to observe both rigid and semirigid residues. Highly mobile residues were detected using 1D and 2D ^1^H─^13^C Insensitive Nuclei Enhanced by Polarization Transfer (INEPT) spectra, as well as ^1^H─^15^N INEPT spectra.

To determine the secondary structure of both P2R tau fibrils and sequentially assign the ^15^N and ^13^C chemical shifts of the rigid core, we measured 2D ^13^C─^13^C and ^15^N─^13^C dipolar correlation spectra and 3D NCACX, NCOCX, and CONCA correlation spectra. To obtain long-range distance constraints for structure calculation, we conducted 3D CCC experiments. These 2D and 3D dipolar correlation experiments required a total of 4 weeks of signal-averaging per sample. ^15^N─^13^C polarization transfer used the SPECIFIC cross-polarization (SPEC-CP) sequence (*39*). The BlackFox crossed-coil dual-resonator low-E coil (*40*) has higher radiofrequency (rf) field homogeneity than solenoid coils; hence, it gives a high SPEC-CP efficiency of ~50% relative to the ^1^H─^13^C CP spectrum. ^13^C spin diffusion was achieved using the Combined-Driven (CORD) sequence (*41*). A ^13^C mixing time of 82 ms was used for the NCACX and NCOCX experiments. These 2D and 3D spectra were measured at 10.5- or 14-kHz MAS. Typical rf field strengths were 71 to 90 kHz for ^1^H, 30 to 50 kHz for ^13^C, and 25 to 40 kHz for ^15^N.

Inter-residue distance constraints were obtained using 3D CCC experiments. For the 24°C fibril, the spectrum was measured using a short ^1^H─^13^C CP contact time of 170 μs to selectively detect the most rigid residues. A DREAM sequence of 2.5 ms was applied to transfer the Cα polarization to Cβ between the first and second ^13^C evolution periods (*42*). The ^13^C rf carrier was placed at ~55 ppm for the DREAM mixing period. A 400-ms CORD mixing period was applied between the second and third dimensions to detect long-range correlations. For the 12°C fibril, the 3D CCC spectrum was measured using a short CORD mixing time of 23 ms between the first and second ^13^C dimensions to obtain one-bond ^13^C─^13^C cross peaks and 400 ms for the mixing time between the second and third dimensions.

Most 2D and 3D spectra were block-averaged and added in the time domain before Fourier transformation. This block averaging allows the correction of magnetic field drift by adjustment of the rf carrier frequency so that identical resonance offsets were maintained between blocks. Spectra were either processed using QSINE apodization (SSB = 3) to enhance spectral resolution or GM apodization (LB = −20 Hz and GB = 0.05) to enhance sensitivity. Spectra were first processed in the Topspin software and then exported to the CCPNMR software for chemical shift assignment (*43*). The tetramethylsilane (TMS)–referenced ^13^C chemical shifts were converted to the DSS scale by adding 2.0 ppm to all values and then converted to secondary chemical shifts to predict (ϕ, ψ) torsion angles using the TALOS-N software (*26*).

To investigate the water accessibility of the fibril core, we measured water-edited 2D N-Cα correlation spectra. The experiment starts with a water-selective T_2_ echo consisting of one rotor period before and after a selective ^1^H 180° Gaussian pulse centered at the water ^1^H chemical shift. The Gaussian pulse length was 950 μs, which corresponds to 10 rotor periods under 10.5-kHz MAS. The selected water ^1^H magnetization was transferred to protein protons by chemical exchange and ^1^H spin diffusion (*44*–*46*), then cross-polarized to protein ^15^N, and correlated with ^13^Cα (*39*). When a short ^1^H mixing time of 4 ms was used, only well-hydrated residues manifest substantial intensities. When a long ^1^H mixing time of 100 ms was used, the ^13^C spectra display the same intensity pattern as the unedited 2D N-Cα spectrum, but with half the intensity, indicating that water and protein ^1^H magnetization has equilibrated. The intensity ratios between the 4-ms spectrum (S) and the 100-ms spectrum (S_0_) give information about the relative water accessibilities of the residues. Uncertainties in the S/S_0_ values were propagated from the signal-to-noise ratios of 1D cross sections of the 2D spectra, using empty regions of the spectra as the noise.

### Chemical shift assignment

The 2D and 3D dipolar correlation spectra were sequential assigned following standard protocols. 2D CC, 2D NCα, and 3D NCACX spectra were first analyzed to identify the residue types of all rigid and semirigid residues, and, then, the Cα and CO chemical shifts were used in conjunction with 3D NCOCX and CONCA spectra for sequential assignment. A composite metric of the secondary chemical shift difference between Cβ and Cα, Δδ_Cβ_ − Δδ_Cα_ ≡ (δ_Cβ,expt_ − δ_Cβ,coil_) − (δ_Cα,expt_ − δ_Cα,coil_), is used to define the β strand segments. Positive Δδ_Cβ_ − Δδ_Cα_ values denote β strand segments, while negative values indicate α-helical segments. Chemical shift differences between the 24° and 12°C P2R tau fibrils are calculated as root mean square deviations of the Cα, Cβ, and CO chemical shifts.

$$\mathrm{CSP}=\sqrt{[{\left(\delta_{C\alpha ,\mathrm{fast}}-\delta_{C\alpha ,\mathrm{slow}}\right)}^{2}+{\left(\delta_{C\beta ,\mathrm{fast}}-\delta_{C\beta ,\mathrm{slow}}\right)}^{2}+{\left(\delta_{CO,\mathrm{fast}}-\delta_{CO,\mathrm{slow}}\right)}^{2}]/3}$$ (1)

### Structure calculation of P2R tau fibril cores

The rigid-core structures of the two P2R tau fibrils were determined using XPLOR-NIH hosted on the NMRBox computing platform (*47*). For the 24°C fibril, five monomers of the protein (residues 274 to 325) were first aligned along the *z* axis with a 20-Å spacing between monomers. The noncrystallographic symmetry potential PostDiffPot was used to restrain the monomers to be structurally identical. Fictitious Nuclear Overhauser Effects (NOEs) between the Cα atoms of the same residue of adjacent chains were used and set to 4.8 ± 0.1 Å. An artificial residual dipolar coupling (rdcPot) potential was applied to the Cα atoms in adjacent chains to align the monomers along the *z* axis. A total of 42 pairs of (ϕ, ψ) angles were inputted (Table 1), and statistical torsion angle database potential (TorsionDB), residue affinity potential, and implicit hydrogen bond database potential (hbond) were applied to each monomer. A total of 103 medium-range correlations (4 > *i* − *j* > 1) and 36 long-range correlations (*i* − *j* ≥ 4) were used as intramolecular distance constraints and were given an upper bound of 8.5 Å. NOE potentials were always applied with the “soft” flag.

For the 12°C P2R fibrils, 5 × 2 monomers of the protein (residues 264 to 326) were first aligned along the *z* axis with a 20-Å spacing between monomers. The noncrystallographic symmetry potential PostDiffPot was used to restrain the monomers to be structurally identical. Fictitious NOEs between the Cα atoms of the same residue of adjacent chains were used and set to 4.8 ± 0.1 Å. An artificial residual dipolar coupling (rdcPot) potential was applied to the Cα atoms in adjacent chains to align the monomers along the *z* axis. An additional 2_1_ screw symmetry was applied with DistSymmPot potential to restrain the monomers in the two stacks, which is so far the main symmetry observed for all brain-extracted dimeric tau fibrils (*8*, *15*). A total of 49 pairs of (ϕ, ψ) angles were inputted, and statistical torsion angle database potential (TorsionDB), residue affinity potential, and implicit hydrogen bond database potential (hbond) were applied to each monomer. Nineteen long-range correlations (*i − j* ≥ 4) were used as intramolecular distance constraints, among which three long-range constraints were explicitly set as dimer interface contacts. NOE potentials were always applied with the soft flag.

Structure calculation in XPLOR-NIH consisted of minimization, simulated annealing, and refinement of the protein. In the minimization step, three extended fibrils made of 5 or 10 copies of P2R tau each were minimized in torsion angle space, first for 2000 steps with only standard bond length, bond angle, and improper angle potentials, then for 5000 steps with these constraints, as well as nonbonded repulsion, PosDiffPot, and the Cα-Cα artificial distance restraints. In the simulated annealing step, 1000 independent XPLOR-NIH runs were performed using high-temperature torsion angle dynamics at 5000 K for 10,000 steps, followed by simulated annealing to 20 K in 20-K decrements with 500 steps of torsion angle dynamics at each temperature. Final energy minimizations were performed in torsion angle and Cartesian coordinates. During all calculations, standard XPLOR-NIH BOND, ANGL, IMPR parameters were used to set the bond lengths and bond angles.

## Notes

View/request a protocol for this paper from *Bio-protocol*.

## References

[1] Y. Wang, E. Mandelkow, Tau in physiology and pathology. Nat. Rev. Neurosci. 17, 5–21 (2016). 26631930 10.1038/nrn.2015.1

[2] V. M. Lee, M. Goedert, J. Q. Trojanowski, Neurodegenerative tauopathies. Annu. Rev. Neurosci. 24, 1121–1159 (2001). 11520930 10.1146/annurev.neuro.24.1.1121

[3] T. Kimura, H. Hatsuta, M. Masuda-Suzukake, M. Hosokawa, K. Ishiguro, H. Akiyama, S. Murayama, M. Hasegawa, S. I. Hisanaga, The abundance of nonphosphorylated tau in mouse and human tauopathy brains revealed by the use of phos-tag method. Am. J. Pathol. 186, 398–409 (2016). 26687814 10.1016/j.ajpath.2015.10.009

[4] C. M. Wischik, M. Novak, P. C. Edwards, A. Klug, W. Tichelaar, R. A. Crowther, Structural characterization of the core of the paired helical filament of Alzheimer disease. Proc. Natl. Acad. Sci. U.S.A. 85, 4884–4888 (1988). 2455299 10.1073/pnas.85.13.4884 PMC280541

[5] R. A. Crowther, Straight and paired helical filaments in Alzheimer disease have a common structural unit. Proc. Natl. Acad. Sci. U.S.A. 88, 2288–2292 (1991). 1706519 10.1073/pnas.88.6.2288 PMC51216

[6] S. Wegmann, I. D. Medalsy, E. Mandelkow, D. J. Müller, The fuzzy coat of pathological human Tau fibrils is a two-layered polyelectrolyte brush. Proc. Natl. Acad. Sci. U.S.A. 110, E313–E321 (2013). 23269837 10.1073/pnas.1212100110 PMC3557036

[7] M. Goedert, R. Jakes, M. G. Spillantini, M. Hasegawa, M. J. Smith, R. A. Crowther, Assembly of microtubule-associated protein tau into Alzheimer-like filaments induced by sulphated glycosaminoglycans. Nature 383, 550–553 (1996). 8849730 10.1038/383550a0

[8] A. W. P. Fitzpatrick, B. Falcon, S. He, A. G. Murzin, G. Murshudov, H. J. Garringer, R. A. Crowther, B. Ghetti, M. Goedert, S. H. W. Scheres, Cryo-EM structures of tau filaments from Alzheimer’s disease. Nature 547, 185–190 (2017). 28678775 10.1038/nature23002 PMC5552202

[9] H. Wesseling, W. Mair, M. Kumar, C. N. Schlaffner, S. J. Tang, P. Beerepoot, B. Fatou, A. J. Guise, L. Cheng, S. Takeda, J. Muntel, M. S. Rotunno, S. Dujardin, P. Davies, K. S. Kosik, B. L. Miller, S. Berretta, J. C. Hedreen, L. T. Grinberg, W. W. Seeley, B. T. Hyman, H. Steen, J. A. Steen, Tau PTM profiles identify patient heterogeneity and stages of Alzheimer’s disease. Cell 183, 1699–1713.e13 (2020). 33188775 10.1016/j.cell.2020.10.029 PMC8168922

[10] S. Dujardin, C. Commins, A. Lathuiliere, P. Beerepoot, A. R. Fernandes, T. V. Kamath, M. B. De Los Santos, N. Klickstein, D. L. Corjuc, B. T. Corjuc, P. M. Dooley, A. Viode, D. H. Oakley, B. D. Moore, K. Mullin, D. Jean-Gilles, R. Clark, K. Atchison, R. Moore, L. B. Chibnik, R. E. Tanzi, M. P. Frosch, A. Serrano-Pozo, F. Elwood, J. A. Steen, M. E. Kennedy, B. T. Hyman, Tau molecular diversity contributes to clinical heterogeneity in Alzheimer’s disease. Nat. Med. 26, 1256–1263 (2020). 32572268 10.1038/s41591-020-0938-9 PMC7603860

[11] B. Falcon, W. Zhang, A. G. Murzin, G. Murshudov, H. J. Garringer, R. Vidal, R. A. Crowther, B. Ghetti, S. H. W. Scheres, M. Goedert, Structures of filaments from Pick’s disease reveal a novel tau protein fold. Nature 561, 137–140 (2018). 30158706 10.1038/s41586-018-0454-y PMC6204212

[12] B. Falcon, W. Zhang, M. Schweighauser, A. G. Murzin, R. Vidal, H. J. Garringer, B. Ghetti, S. H. W. Scheres, M. Goedert, Tau filaments from multiple cases of sporadic and inherited Alzheimer’s disease adopt a common fold. Acta Neuropathol. 136, 699–708 (2018). 30276465 10.1007/s00401-018-1914-z PMC6208733

[13] B. Falcon, J. Zivanov, W. Zhang, A. G. Murzin, H. J. Garringer, R. Vidal, R. A. Crowther, K. L. Newell, B. Ghetti, M. Goedert, S. H. W. Scheres, Novel tau filament fold in chronic traumatic encephalopathy encloses hydrophobic molecules. Nature 568, 420–423 (2019). 30894745 10.1038/s41586-019-1026-5 PMC6472968

[14] W. Zhang, A. Tarutani, K. L. Newell, A. G. Murzin, T. Matsubara, B. Falcon, R. Vidal, H. J. Garringer, Y. Shi, T. Ikeuchi, S. Murayama, B. Ghetti, M. Hasegawa, M. Goedert, S. H. W. Scheres, Novel tau filament fold in corticobasal degeneration. Nature 580, 283–287 (2020). 32050258 10.1038/s41586-020-2043-0 PMC7148158

[15] Y. Shi, W. Zhang, Y. Yang, A. G. Murzin, B. Falcon, A. Kotecha, M. van Beers, A. Tarutani, F. Kametani, H. J. Garringer, R. Vidal, G. I. Hallinan, T. Lashley, Y. Saito, S. Murayama, M. Yoshida, H. Tanaka, A. Kakita, T. Ikeuchi, A. C. Robinson, D. M. A. Mann, G. G. Kovacs, T. Revesz, B. Ghetti, M. Hasegawa, M. Goedert, S. H. W. Scheres, Structure-based classification of tauopathies. Nature 598, 359–363 (2021). 34588692 10.1038/s41586-021-03911-7 PMC7611841

[16] A. J. Dregni, H. K. Wang, H. F. Wu, P. Duan, J. Jin, W. F. DeGrado, M. Hong, Inclusion of the C-terminal domain in the β-sheet core of heparin-fibrillized three-repeat Tau protein revealed by solid-state nuclear magnetic resonance spectroscopy. J. Am. Chem. Soc. 143, 7839–7851 (2021). 33983722 10.1021/jacs.1c03314 PMC8283780

[17] T. Kampers, P. Friedhoff, J. Biernat, E. M. Mandelkow, E. Mandelkow, RNA stimulates aggregation of microtubule-associated protein tau into Alzheimer-like paired helical filaments. FEBS Lett. 399, 344–349 (1996). 8985176 10.1016/s0014-5793(96)01386-5

[18] A. J. Dregni, V. S. Mandala, H. Wu, M. R. Elkins, H. K. Wang, I. Hung, W. F. DeGrado, M. Hong, In vitro 0N4R tau fibrils contain a monomorphic β-sheet core enclosed by dynamically heterogeneous fuzzy coat segments. Proc. Natl. Acad. Sci. U.S.A. 116, 16357–16366 (2019). 31358628 10.1073/pnas.1906839116 PMC6697781

[19] A. J. Dregni, P. Duan, M. Hong, Hydration and dynamics of full-length Tau amyloid fibrils investigated by solid-state nuclear magnetic resonance. Biochemistry 59, 2237–2248 (2020). 32453948 10.1021/acs.biochem.0c00342 PMC7720860

[20] W. Zhang, B. Falcon, A. G. Murzin, J. Fan, R. A. Crowther, M. Goedert, S. H. Scheres, Heparin-induced tau filaments are polymorphic and differ from those in Alzheimer’s and Pick’s diseases. eLife 8, e43584 (2019). 30720432 10.7554/eLife.43584 PMC6375701

[21] N. El Mammeri, A. J. Dregni, P. Duan, H. K. Wang, M. Hong, Microtubule-binding core of the Tau protein. Sci. Adv. 8, eabo4459 (2022). 35857846 10.1126/sciadv.abo4459 PMC9299549

[22] A. J. Dregni, P. Duan, H. Xu, L. Changolkar, N. El Mammeri, V. M. Lee, M. Hong, Fluent molecular mixing of Tau isoforms in Alzheimer’s disease neurofibrillary tangles. Nat. Commun. 13, 2967 (2022). 35624093 10.1038/s41467-022-30585-0 PMC9142584

[23] N. Gustke, B. Trinczek, J. Biernat, E. M. Mandelkow, E. Mandelkow, Domains of tau protein and interactions with microtubules. Biochemistry 33, 9511–9522 (1994). 8068626 10.1021/bi00198a017

[24] E. M. Mandelkow, J. Biernat, G. Drewes, N. Gustke, B. Trinczek, E. Mandelkow, Tau domains, phosphorylation, and interactions with microtubules. Neurobiol. Aging 16, 355–362 (1995). 7566345 10.1016/0197-4580(95)00025-a

[25] Y. Wang, O. Jardetzky, Probability-based protein secondary structure identification using combined NMR chemical-shift data. Protein Sci. 11, 852–861 (2002). 11910028 10.1110/ps.3180102 PMC2373532

[26] Y. Shen, A. Bax, Protein backbone and sidechain torsion angles predicted from NMR chemical shifts using artificial neural networks. J. Biomol. NMR 56, 227–241 (2013). 23728592 10.1007/s10858-013-9741-y PMC3701756

[27] M. R. Sawaya, S. Sambashivan, R. Nelson, M. I. Ivanova, S. A. Sievers, M. I. Apostol, M. J. Thompson, M. Balbirnie, J. J. Wiltzius, H. T. McFarlane, A. Ø. Madsen, C. Riekel, D. Eisenberg, Atomic structures of amyloid cross-beta spines reveal varied steric zippers. Nature 447, 453–457 (2007). 17468747 10.1038/nature05695

[28] M. Chen, N. P. Schafer, P. G. Wolynes, Surveying the energy landscapes of Aβ fibril polymorphism. J. Phys. Chem. B 122, 11414–11430 (2018). 30215519 10.1021/acs.jpcb.8b07364 PMC6713213

[29] Y. Kusumoto, A. Lomakin, D. B. Teplow, G. B. Benedek, Temperature dependence of amyloid beta-protein fibrillization. Proc. Natl. Acad. Sci. U.S.A. 95, 12277–12282 (1998). 9770477 10.1073/pnas.95.21.12277 PMC22822

[30] R. Nelson, M. R. Sawaya, M. Balbirnie, A. Ø. Madsen, C. Riekel, R. Grothe, D. Eisenberg, Structure of the cross-β spine of amyloid-like fibrils. Nature 435, 773–778 (2005). 15944695 10.1038/nature03680 PMC1479801

[31] W. Qiang, K. Kelley, R. Tycko, Polymorph-specific kinetics and thermodynamics of β-amyloid fibril growth. J. Am. Chem. Soc. 135, 6860–6871 (2013). 23627695 10.1021/ja311963f PMC3686096

[32] M. Törnquist, T. C. T. Michaels, K. Sanagavarapu, X. Yang, G. Meisl, S. I. A. Cohen, T. P. J. Knowles, S. Linse, Secondary nucleation in amyloid formation. Chem. Commun. 54, 8667–8684 (2018). 10.1039/c8cc02204f 29978862

[33] J. W. P. Schmelzer, A. S. Abyzov, V. M. Fokin, C. Schick, E. D. Zanotto, Crystallization of glass-forming liquids: Maxima of nucleation, growth, and overall crystallization rates. J. Non. Cryst. Solids 429, 24–32 (2015).

[34] J. S. Pedersen, D. Dikov, J. L. Flink, H. A. Hjuler, G. Christiansen, D. E. Otzen, The changing face of glucagon fibrillation: Structural polymorphism and conformational imprinting. J. Mol. Biol. 355, 501–523 (2006). 16321400 10.1016/j.jmb.2005.09.100

[35] M. D. Mukrasch, M. von Bergen, J. Biernat, D. Fischer, C. Griesinger, E. Mandelkow, M. Zweckstetter, The “jaws” of the tau-microtubule interaction. J. Biol. Chem. 282, 12230–12239 (2007). 17307736 10.1074/jbc.M607159200

[36] A. Savastano, G. Jaipuria, L. Andreas, E. Mandelkow, M. Zweckstetter, Solid-state NMR investigation of the involvement of the P2 region in tau amyloid fibrils. Sci. Rep. 10, 21210 (2020). 33273615 10.1038/s41598-020-78161-0 PMC7712923

[37] S. Lövestam, F. A. Koh, B. van Knippenberg, A. Kotecha, A. G. Murzin, M. Goedert, S. H. W. Scheres, Assembly of recombinant tau into filaments identical to those of Alzheimer’s disease and chronic traumatic encephalopathy. eLife 11, e76494 (2022). 35244536 10.7554/eLife.76494 PMC8983045

[38] A. Böckmann, C. Gardiennet, R. Verel, A. Hunkeler, A. Loquet, G. Pintacuda, L. Emsley, B. H. Meier, A. Lesage, Characterization of different water pools in solid-state NMR protein samples. J. Biomol. NMR 45, 319–327 (2009). 19779834 10.1007/s10858-009-9374-3

[39] M. Baldus, A. T. Petkova, J. Herzfeld, R. G. Griffin, Cross polarization in the tilted frame: Assignment and spectral simplification in heteronuclear spin systems. Mol. Phys. 95, 1197–1207 (1998).

[40] S. A. McNeill, P. L. Gor'kov, K. Shetty, W. W. Brey, J. R. Long, A low-E magic angle spinning probe for biological solid state NMR at 750 MHz. J. Magn. Reson. 197, 135–144 (2009). 19138870 10.1016/j.jmr.2008.12.008 PMC2659328

[41] G. Hou, S. Yan, S. Sun, Y. Han, I. J. Byeon, J. Ahn, J. Concel, A. Samoson, A. M. Gronenborn, T. Polenova, Spin diffusion driven by R-symmetry sequences: Applications to homonuclear correlation spectroscopy in MAS NMR of biological and organic solids. J. Am. Chem. Soc. 133, 3943–3953 (2011). 21361320 10.1021/ja108650x PMC3148607

[42] R. Verel, M. Ernst, B. H. Meier, Adiabatic dipolar recoupling in solid-state NMR: The DREAM scheme. J. Magn. Reson. 150, 81–99 (1998). 10.1006/jmre.2001.2310 11330986

[43] S. P. Skinner, R. H. Fogh, W. Boucher, T. J. Ragan, L. G. Mureddu, G. W. Vuister, CcpNmr AnalysisAssign: A flexible platform for integrated NMR analysis. J. Biomol. NMR 66, 111–124 (2016). 27663422 10.1007/s10858-016-0060-y PMC5095159

[44] J. K. Williams, M. Hong, Probing membrane protein structure using water polarization transfer solid-state NMR. J. Magn. Reson. 247, 118–127 (2014). 25228502 10.1016/j.jmr.2014.08.007 PMC4398059

[45] T. Wang, H. Jo, W. F. DeGrado, M. Hong, Water distribution, dynamics, and interactions with Alzheimer’s β-amyloid fibrils investigated by solid-state NMR. J. Am. Chem. Soc. 139, 6242–6252 (2017). 28406028 10.1021/jacs.7b02089 PMC5808936

[46] O. C. Andronesi, M. von Bergen, J. Biernat, K. Seidel, C. Griesinger, E. Mandelkow, M. Baldus, Characterization of Alzheimer’s-like paired helical filaments from the core domain of tau protein using solid-state NMR spectroscopy. J. Am. Chem. Soc. 130, 5922–5928 (2008). 18386894 10.1021/ja7100517

[47] M. W. Maciejewski, A. D. Schuyler, M. R. Gryk, I. I. Moraru, P. R. Romero, E. L. Ulrich, H. R. Eghbalnia, M. Livny, F. Delaglio, J. C. Hoch, NMRbox: A resource for biomolecular NMR computation. Biophys. J. 112, 1529–1534 (2017). 28445744 10.1016/j.bpj.2017.03.011 PMC5406371

