# Supplementary material for: Amyloid fibril structures of tau: Conformational plasticity of the second microtubule-binding repeat
Source: Sci Adv. 2023 Jul 14;9(28):eadh4731. doi: 10.1126/sciadv.adh4731 (PMC10348678; doi:10.1126/sciadv.adh4731)
Supplement: Supplementary file 1 — Figs. S1 to S8 Tables S1 to S9 [file sciadv.adh4731_sm.pdf]

Science Advances
AAAS

Supplementary Materials for

Amyloid fibril structures of tau: Conformational plasticity of the second microtubule-binding repeat

Nadia El Mammeri *et al.*

Corresponding author: Mei Hong, meihong@mit.edu

Sci. Adv. **9**, eadh4731 (2023)
DOI: 10.1126/sciadv.adh4731

This PDF file includes:

Figs. S1 to S8
Tables S1 to S9

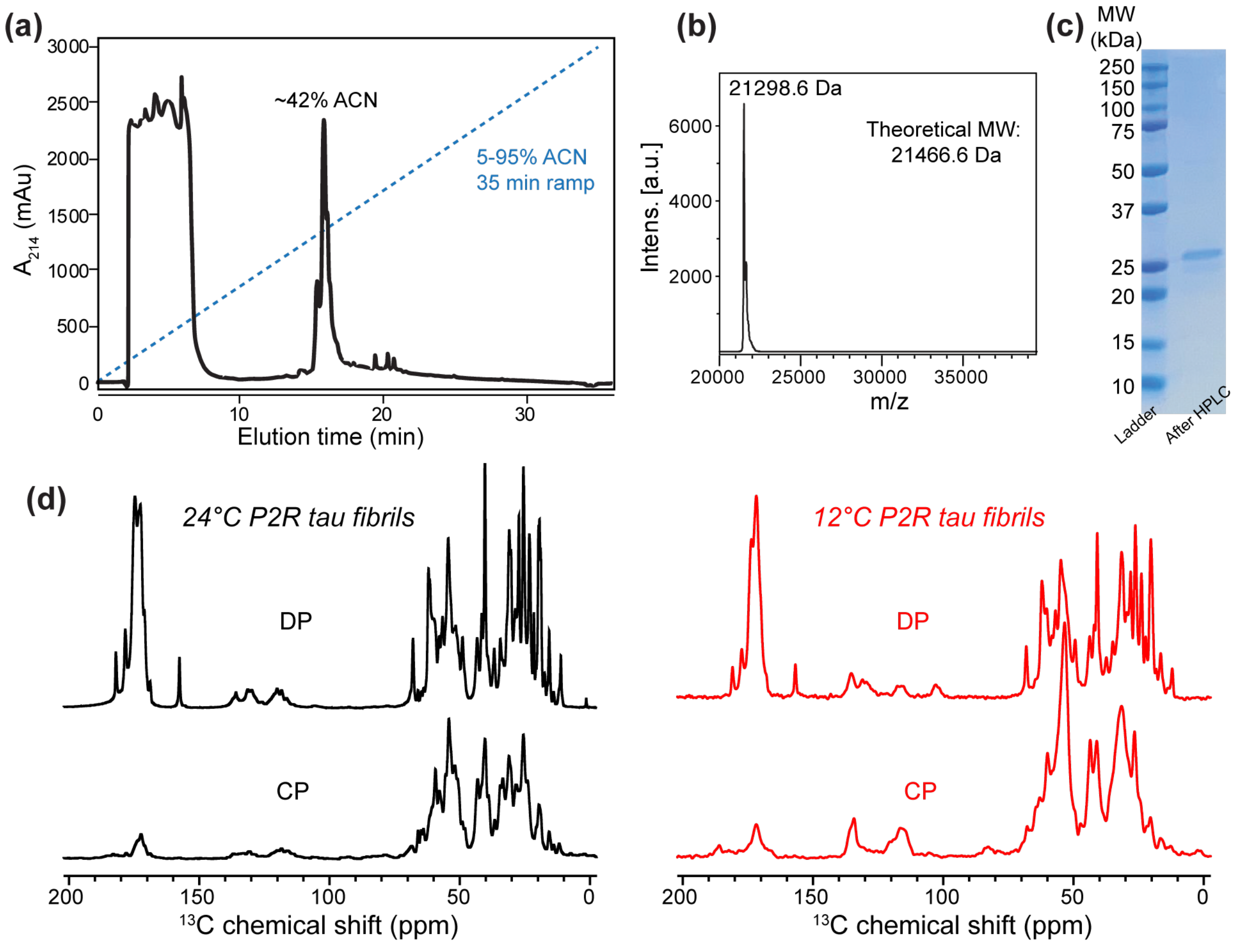

Figure S1. Purification and characterization of P2R tau. (**a**) HPLC chromatogram of P2R tau monomers. (**b**) MALDI mass spectrum. The difference between the measured and expected molecular weight is due to instrument calibration, as tested on model proteins. (**c**) SDS-PAGE gel of P2R tau after HPLC purification. (**d**) 1D ^{13}C NMR spectra of heparin-induced P2R tau fibrils obtained at 24˚C (left) and at 12˚C (right). DP spectra were measured with a recycle delay of 3 s and show roughly quantitative intensities of all residues, while CP spectra were measured using a 70 µs contact time and show the signals of the most rigid residues. The low-temperature fibril has higher CP intensities relative to the DP intensities as compared to the high-temperature fibril, indicating that the low-temperature fibrils have a larger rigid core.

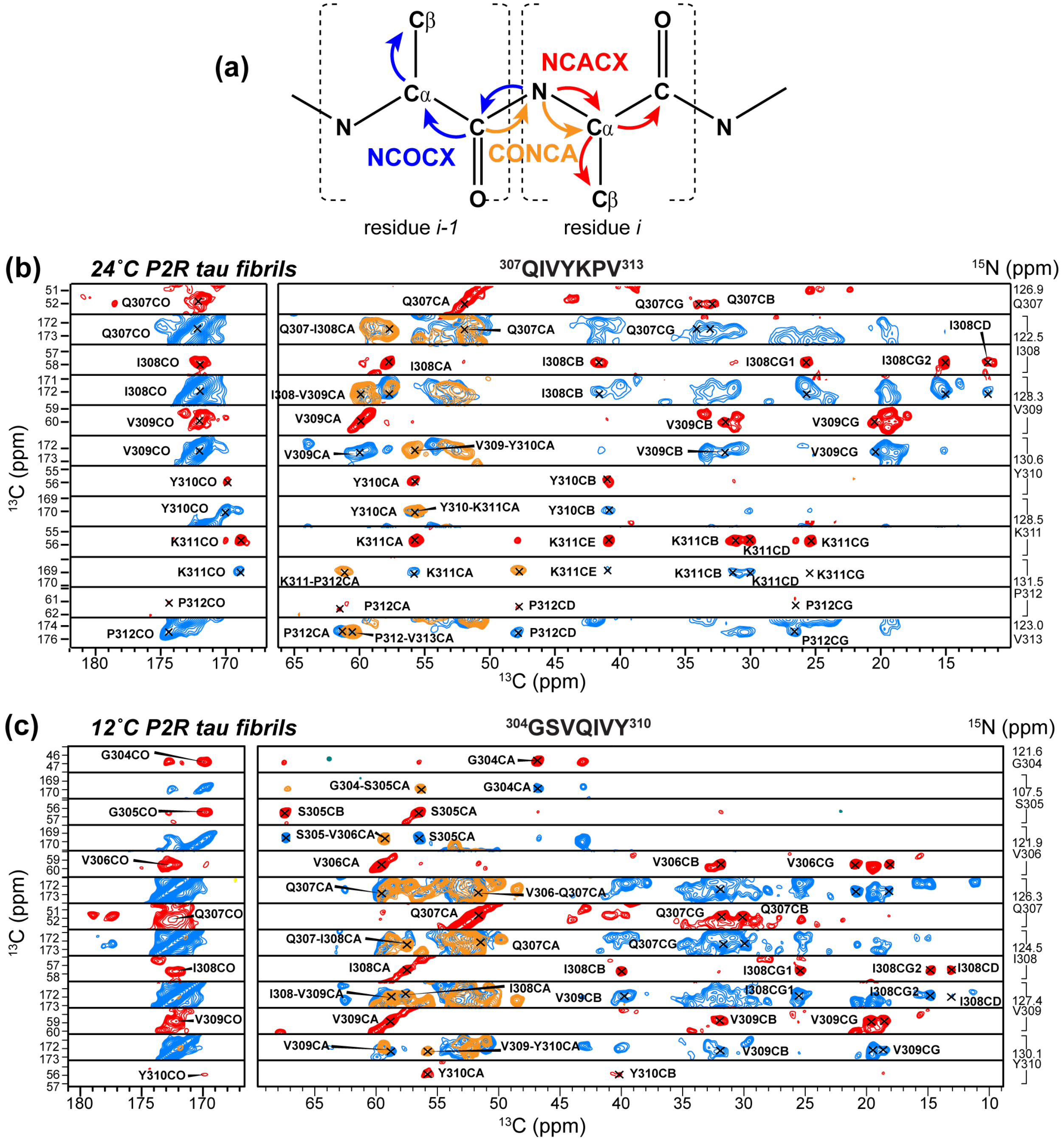

Figure S2. Representative strips of the 3D correlation spectra for resonance assignment of P2R tau fibrils. (**a**) Schematic diagram of the 3D NCACX (red), NCOCX (blue) and CONCA (orange) correlation scheme. (**b**) 3D spectral strips for the ^{307}QVIYLPV^{313} segment of the 24˚C fibril. (**b**) 3D spectral strips for the ^{304}GSVQIVY^{310} segment of the 12˚C fibril.

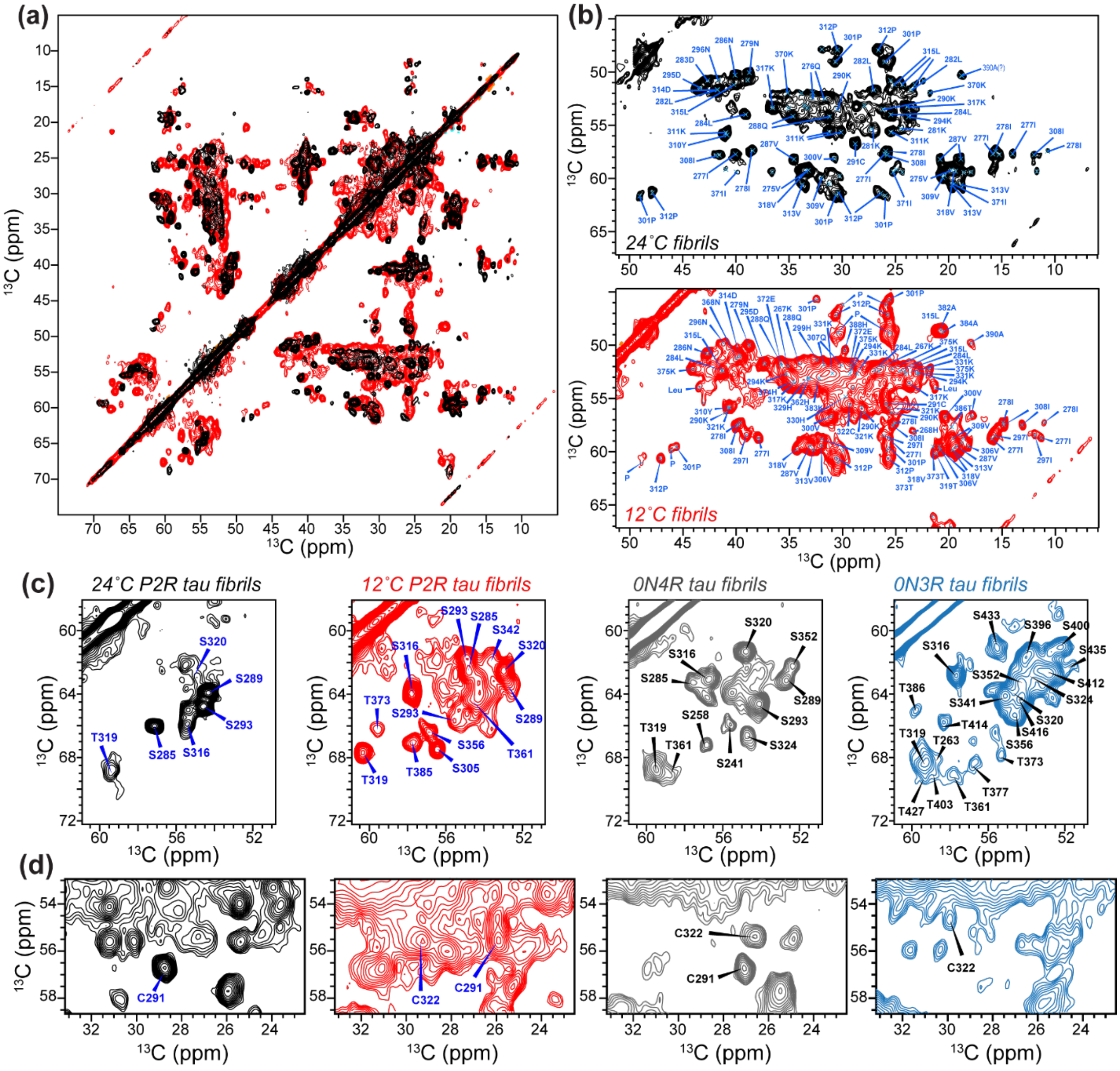

Figure S3. Comparison of the 2D CC spectra of 24˚C P2R tau (black), 12˚C P2R tau (red), full-length 0N4R tau (gray), and full-length 0N3R tau (blue). All four samples were fibrillized using heparin. The spectra were measured with 23 ms ^{13}C spin diffusion. (**a**) Overview of the aliphatic region of the spectra of the two P2R tau fibrils. (**b**) Expanded Cα to sidechain correlation spectra of the spectra of the two P2R tau fibrils with assignment indicated. (**c**) Ser/Thr region of the 2D CC spectra. (**d**) Cys region of the 2D CC spectra.

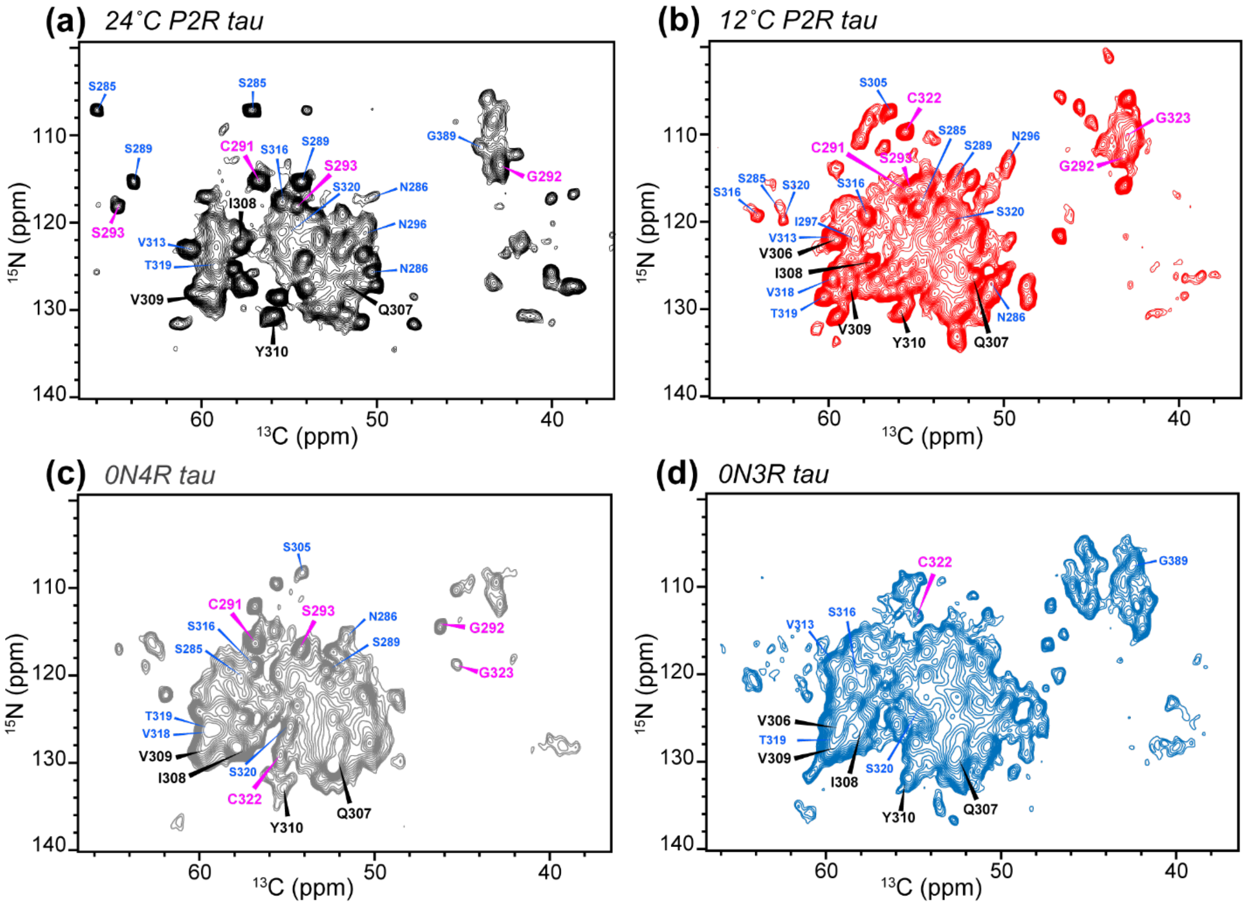

Figure S4. Comparison of 2D NC spectra of four tau fibrils. (**a**) The 24˚C P2R tau fibril. (**b**) The 12˚C P2R tau fibril. (**c**) 0N4R tau fibril (*18*). (**d**) 0N3R tau fibril (*16*). Assignments for selected residues are shown. The R3 hexapeptide residues (assigned in black) have conserved chemical shifts among the four samples, indicating similar conformations, while the two CGS motifs (assigned in pink) have very different chemical shifts, indicating that these two CGS motifs are hotspots of conformational changes.

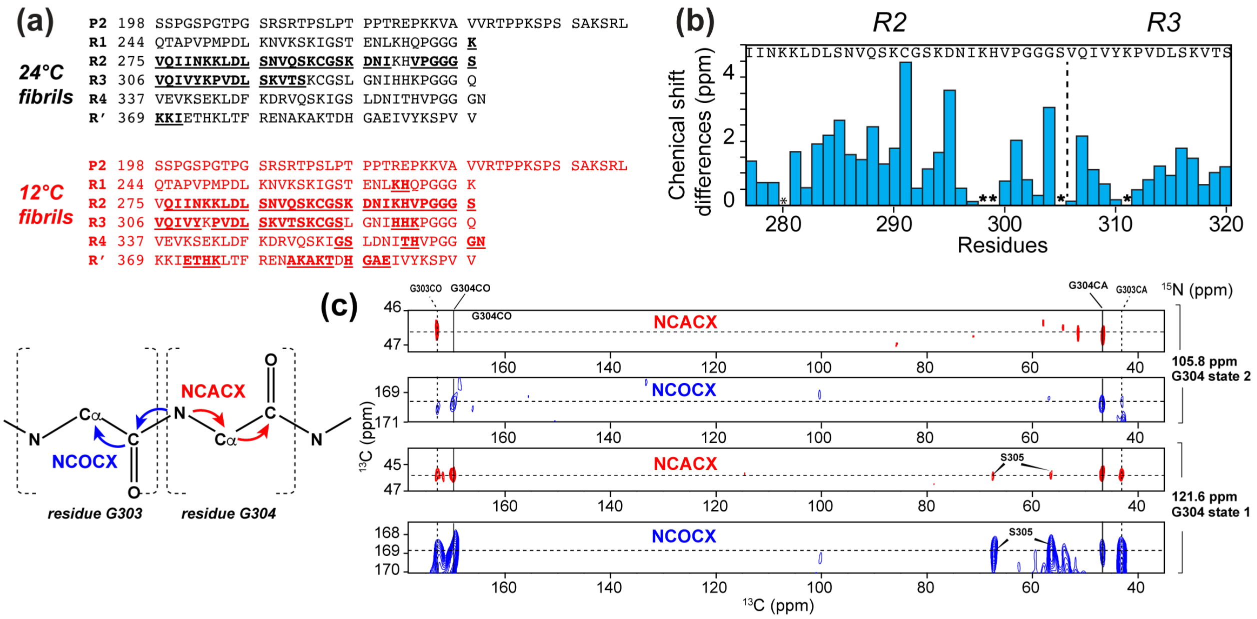

Figure S5. Chemical shifts of P2R tau fibrils. (**a**) Assigned residues (bold and underlined) in the amino acid sequence of P2R tau for the 24˚C fibril (black) and 12˚C fibril (red). (**b**) Chemical shift differences between the 12˚C and 24˚C fibrils, calculated as the root mean square deviations of the Cα, Cβ and CO chemical shifts. Residues for which assignment is missing in one of the fibrils are indicated with an asterisk. (**c**) Representative strips of the 3D NCACX and NCOCX spectra for resonance assignment of the two states of Gly304 in the 12˚C fibril. State 1 of Gly304 has strong intensities but an unusually downfield ^{15}N chemical shift of 121.6 ppm. State 2 has weak intensities and has a typical glycine ^{15}N chemical shift of 105.8 ppm.

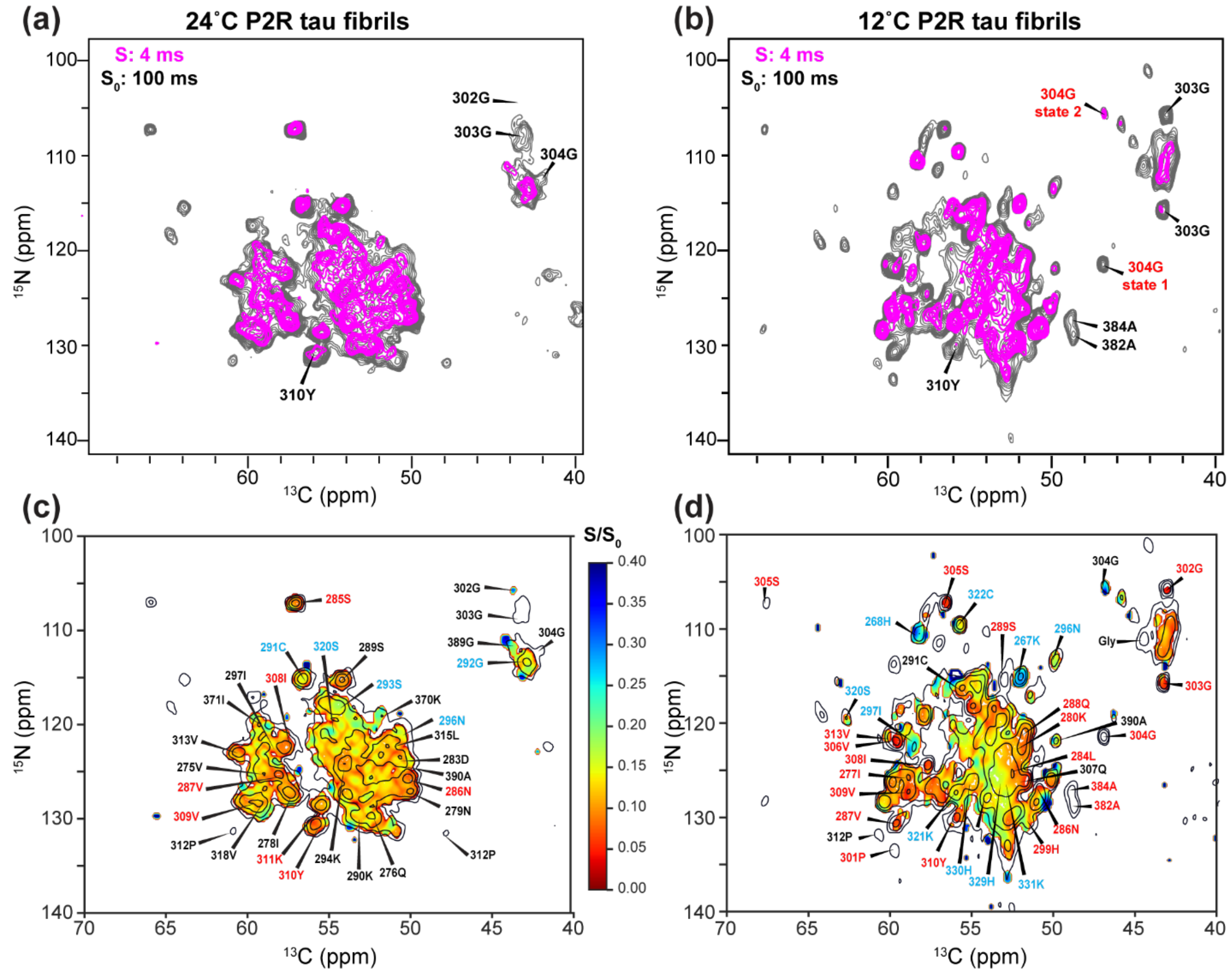

Figure S6. Water-edited NMR spectra of P2R tau fibrils indicate site-specific water accessibilities of the protein. (**a**) 2D NC spectrum of the 24˚C fibril, measured using 4 ms (pink) and 100 ms (grey) water ^{1}H magnetization transfer to the protein. Selected assignments are indicated. (**b**) 2D NC spectrum of the 12˚C fibril measured with 4 ms and 100 ms ^{1}H mixing times for water magnetization transfer to the protein. Note that G304 has two correlation peaks, with the major peak being water inaccessible (state 1) and the minor peak being water accessible (state 2). (**c**) 2D NC hydration map of the 24˚C fibril. Contour levels indicate the intensity ratios between 4 ms spectrum (S) and the 100 ms (S_0) spectrum. Selected assignments are shown in red for the most water-inaccessible residues, cyan for the most water-accessible residues, and black for some of the other residues. (**d**) 2D NC hydration map of the 12˚C fibril.

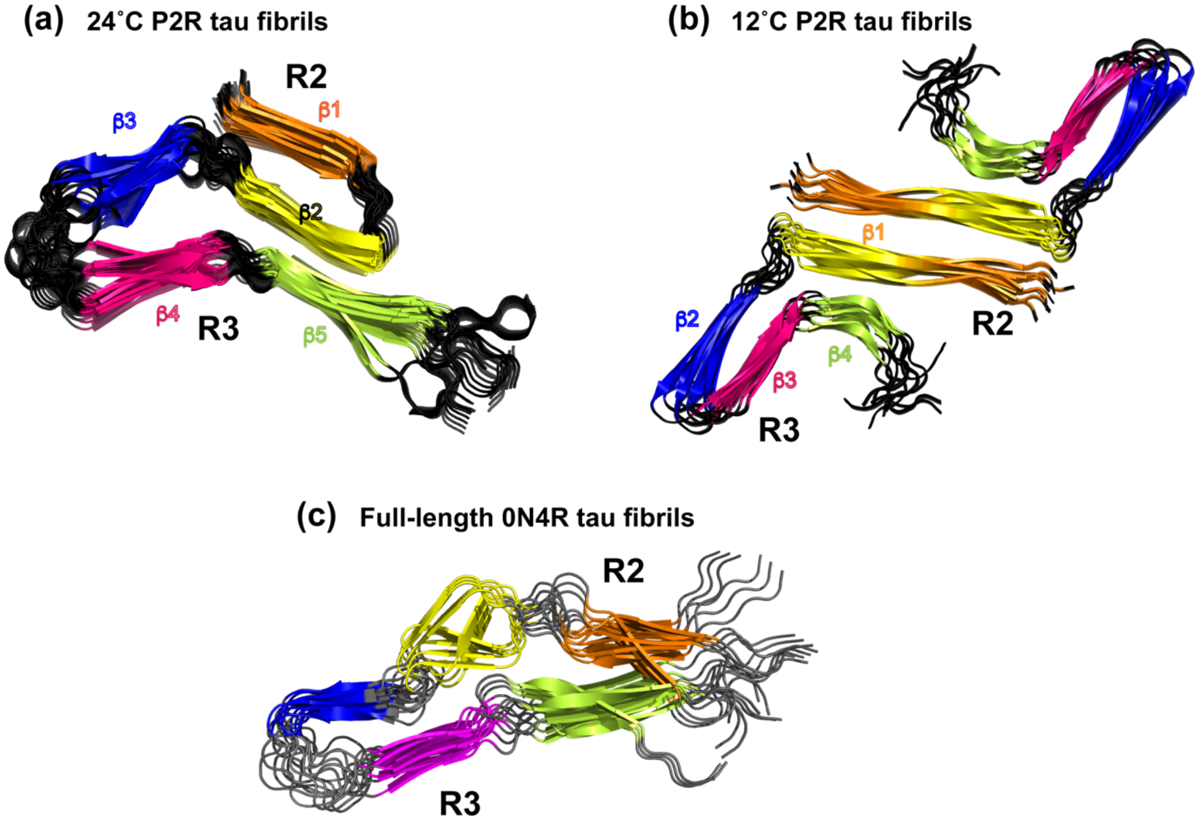

Figure S7. Comparison of the three-dimensional folds of P2R tau fibrils with full-length 0N4R tau fibrils. (**a**) Lowest-energy ensemble of ten structural models of the 24˚C P2R tau fibril. The N-terminal half of R2 forms a β-arch. (**b**) Lowest-energy ensemble of ten structural models of the 12˚C P2R tau fibril. The N-terminal half of R2 adopts a contiguous β-strand that lies at the interface of two protofilaments. (**c**) Lowest-energy ensemble of five structural models of full-length 0N4R tau fibril (*18*).

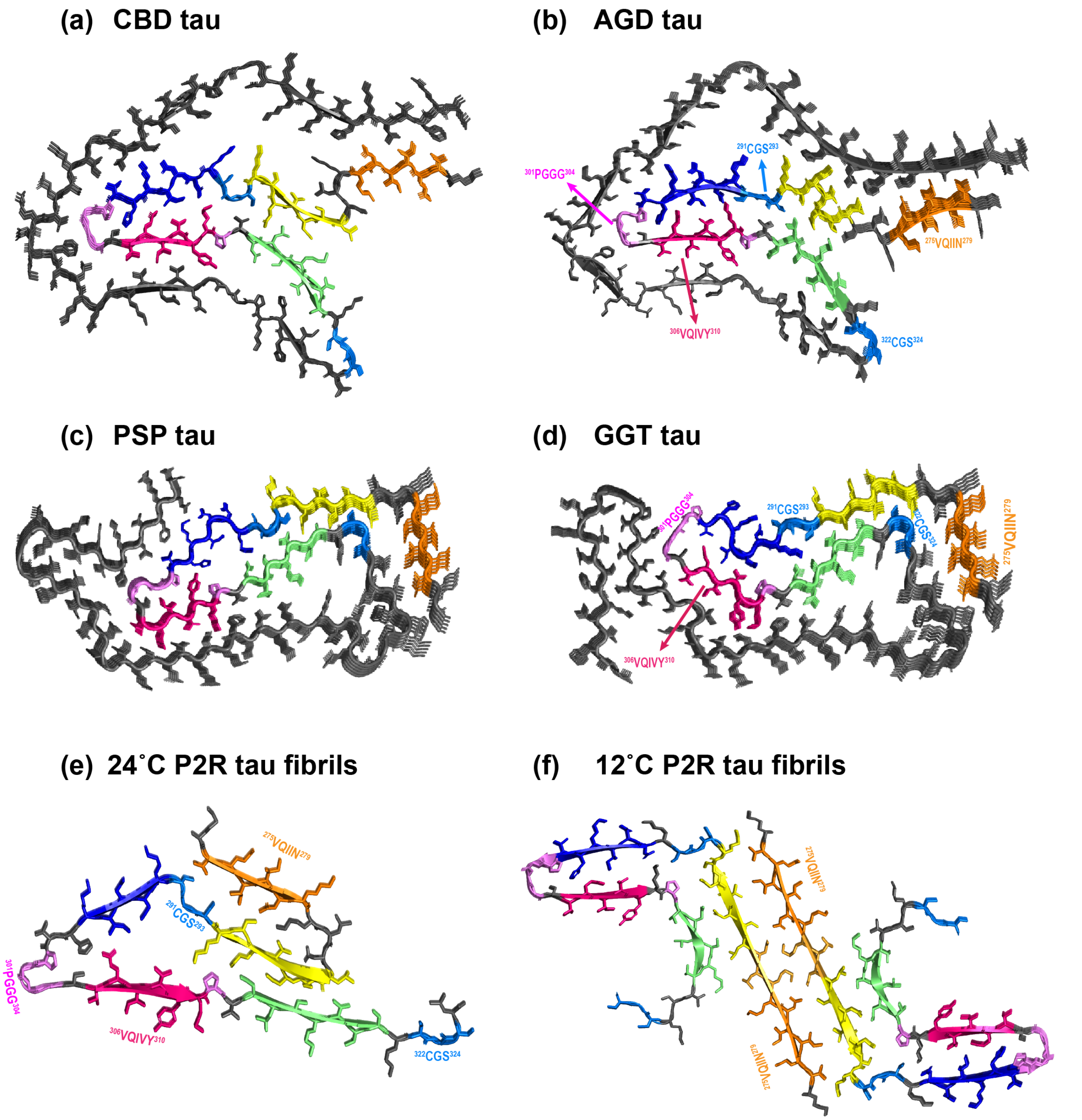

Figure S8. Comparison of the fibril core structures of brain-derived *ex vivo* 4R tau and heparin-induced P2R tau (*14, 15*). (**a**) CryoEM structure of CBD type 1 tau fibrils (*14*). (**b**) CryoEM structure of AGD type 1 tau fibrils (*14*). (**c**) CryoEM structure of PSP tau fibrils. (**d**) CryoEM structure of GGT tau fibrils. (**e**) Ambient-temperature P2R tau fibrils induced with heparin at 24˚C. (**f**) Low-temperature P2R tau fibrils induced with heparin at 12˚C. In all structures, the two hexapeptides of R2 and R3 are colored in orange, the ^{291}CGS^{293} and ^{322}CGS^{324} motifs are in cyan, and P312 and ^{301}PGGG^{304} are colored in magenta. All models are oriented with the ^{300}VPGGG^{304} segment on the left.

Table S1. ^{13}C and ^{15}N chemical shifts (ppm) of immobilized residues in the 24˚C P2R tau fibrils. ^{13}C chemical shifts were referenced externally to the adamantane CH_2 chemical shift at 38.48 ppm on the tetramethylsilane scale. ^{15}N chemical shifts were referenced to the ^{15}N peak of N-acetylvaline at 122.00 ppm on the liquid ammonia scale.

Residues	N	Nδ	Nε	C'	Cα	Cβ	Cγ/γ1	Cγ2	Cδ/δ1	Cδ2	Cε/ε1	Cζ
Lys274				173.6								
Val275	125.0			172.1	59.2	33.2	19.7					
Gln276	129.9			172.0	52.5	31.6	32.8		177.5			
Ile277	126.7			171.6	57.7	40.1	26.0	15.8	13.9			
Ile278	127.3			171.1	57.5	38.5	25.8	15.6	10.7			
Asn279	127.1	116.7		170.5	50.1	38.8	174.1					
Lys280	123.2			173.0	52.1	35.4	24.0		28.1			
Lys281	124.0			172.9	55.3	33.7	24.5		27.2			
Leu282	122.4			173.4	51.7	41.8	27.0		23.5			
Asp283	123.6			174.1	50.8	42.6	177.7					
Leu284	127.3			172.4	54.0	39.1	25.4		23.7			
Ser285	107.1			168.7	57.2	66.0						
Asn286	125.7	117.1		170.6	50.3	40.1	173.8					
Val287	125.3			172.3	58.2	34.5	18.8	20.8				
Gln288	124.1		117.0	174.9	54.2	31.1	34.5		178.0			
Ser289	115.3			171.7	54.3	63.9						
Lys290	129.9			173.0	53.4	30.5	26.0					
Cys291	115.2			172.1	56.7	28.7						
Gly292	113.3			170.8	43.0							
Ser293	118.1			172.0	54.6	64.7						
Lys294	124.7			172.6	54.2	32.9	23.9		28.1		40.6	
Asp295	126.8			171.7	51.5	43.4	181.5					
Asn296	121.0			171.7	50.7	41.6	174.9					
Ile297	122.2			171.6	58.7	39.0	24.8	15.6	11.9			
Val300	120.8				58.0	30.7	20.5	19.0				
Pro301	134.4			173.5	61.6	30.6	26.0		49.0			
Gly302	105.8			170.1	43.7							
Gly303	108.6			173.1	43.4							
Gly304	112.1			170.2	42.0							
Ser305	114.6											
Val306						31.8						
Gln307	126.9		116.9	172.4	52.0	33.8	32.8		178.6			
Ile308	122.5			172.3	57.8	41.8	25.7	15.2	11.9			
Val309	128.3			172.2	59.9	32.0	20.5					
Tyr310	130.6			170.0	55.9	40.8	130.5					157.6
Lys311	128.5			169.0	55.7	31.2	25.4		30.1		41.0	
Pro312	131.5			174.5	61.4	30.4	26.6		47.9			
Val313	123.0			172.4	60.7	33.6	18.9	19.6				
Asp314	130.6			171.5	51.7	41.8	179.9					
Leu315	122.1			175.1	51.1	40.3	25.2		23.2	24.3		
Ser316	117.8			170.7	55.5	65.9						
Lys317	121.8			173.2	53.4	36.5	28.7		24.0		40.1	
Val318	128.3			173.6	59.3	33.4	19.3	19.4				
Thr319	124.7			171.5	59.4	69.2		19.9				
Ser320	121.0			172.0	55.0	63.0						
Lys369				173.6								

Lys370	119.0			171.7	51.9	35.1	21.7		
Ile371	119.9				59.4	39.9	25.2	18.6	14.1

Table S2. ^{13}C and ^{15}N chemical shifts of the 12˚C P2R tau fibrils. Italics denote a second set of chemical shifts for S293 and G304.

Residues	N	Nδ	Nε	C'	Cα	Cβ	Cγ/γ1	Cγ2	Cδ/δ1	Cδ2	Cε/ε1	Cζ
Lys267	115.18			170.59	51.95	35.14			26.21			
His268	110.67			172.69	58.14	23.25					134.99	
Gln276				171.65	52.39	31.44	32.85					
Ile277	125.82			171.65	58.74	37.94	25.64	15.91	11.3			
Ile278	126.45			171.74	57.27	39.6	25.05	14.87	11.06			
Asn279	126.03	107.4		171.76	49.98	38.82	173.58					
Lys280	122.69				52.07							
Lys281	127.14			172.93	52.48	33.18	25.27		29.01		44	
Leu282	125.4			173.25	52.37	41.2	27.22		23.05			
Asp283	125.8			171.52	51.53	40.65	178.67					
Leu284	125.04			174.97	52.38	41.34	27.09		24.18			
Ser285	118.26			170.88	54.81	62.65						
Asn286	126.77				50.77	42.76	172.18					
Val287	130.61			173.69	59.5	33.01	19.28					
Gln288	121.35		115.86	173.03	51.65	34.03	35.75		179.16			
Ser289	115.28			173.17	52.88	63.11						
Lys290	125.09			173.96	56.35		25.41		27.96		41.04	
Cys291	116.58			172.85	55.5	25.97						
Gly292	112.59			171.69	43.14							
Ser293	116.09			172.67	55.41	61.83						
Ser293	*115.76*			*172.84*	*54.88*	*65.72*						
Lys294	132.92			172.7	52.62	35.21	22.85		29.39			
Asp295	122.02			173.15	51.74	37.35	180.51					
Asn296	113.3			175.1	49.8	41.37						
Ile297	121.83				58.47	39.07	26.1	15.65	11.92			
Lys298				171.03	52.58							
His299	130.43			172.51	51.4	32.4			114.8		135.41	
Val300	127.9			169.2	56.7	31.1	17.85	20.37				
Pro301	133.38			175.6	59.55	32.47	25.62		45.63			
Gly302	105.91			171.35	42.96							
Gly303	115.8			172.65	43.05							
Gly304	121.62			172.53	46.77							
Gly304	*105.8*			*172.84*	*46.76*							
Ser305	107.45			169.77	56.47	67.38						
Val306	121.92			172.64	59.5	31.96	20.86	18.15				
Gln307	126.29		111.68	172.46	51.8	30.06	31.81		177.33			
Ile308	124.47			172.18	57.57	39.91	25.28	14.75	13.08			
Val309	127.43			172.01	58.78	32.02	18.65		19.58			
Tyr310	130.09			169.97	55.83	40.51						
Pro312	131.52			174.93	60.61	30.59	25.73		47.09			
Val313	121.68			173.1	59.81	32.77	19.55					
Asp314	128.42			171.86	50.97	39.77	178.27					
Leu315	129.8			174.97	52.4	41.24	26.59		24.61			
Ser316	119.19			171.4	57.79	63.96						
Lys317	118.16			174.71	53.56	34.52	23.82					
Val318	126.27			173.05	59.8	34.34	20.82	19.64				
Thr319	128.4			171.09	60.25	67.66		21.17				
Ser320	119.68			172.58	53.08	62.59						
Lys321	127.63			172.76	55.84		25.68		28.2		40.62	

Cys322	109.76			172.58	55.67	29.3				
Gly323	109.99				43.13					
Ser324	121.75				53.76	61.95				
His329	126.47			171.31	53.05	33.19		114.66	134.41	
His330	126.39	-	-	170.61	54.84	29.8	-	114.14	132.2	-
Lys331	126.42	-	-	173.09	52.59	31.04	23.93	23.13	28.66	-
Gly355	119.14	-	-	172.8	46.29	-	-	-	-	-
Ser356	111.36	-	-	169.27	56.82	66.36	-	-	-	-
Thr361	117.93	-	-	171.31	54.69	64.46	-	-	-	-
His362	123.8	-	-	172.02	53.11	33.2	134.97	114.55	135.84	-
Gly367	106.94	-	-	172.23	45.75	-	-	-	-	-
Asn368	117.03	-	-	-	51.19	40.11	-	-	-	-
Glu372	123.09	-	-	174.19	51.81	29.21	35.82	181.68	-	-
Thr373	113.91	-	-	174.26	59.59	66.19	21.5	-	-	-
His374	125.72	-	-	171.58	53.44	34.61	-	113.8	134.76	-
Lys375	126.04	-	-	-	52.27	29.14	21.86	25.34	44.05	-
Ala382	128.75	-	-	173.01	48.63	20.78	-	-	-	-
Lys383	118.36	-	-	172.36	53.08	32.15	-	-	-	-
Ala384	127.07	-	-	173.2	48.71	20.64	-	-	-	-
Lys385	119.63	-	-	174.91	53.63	31.2	-	-	-	-
Thr386	108.51	-	-	172.6	57.7	67.06	19.74	-	-	-
His388	128.23	-	-	-	50.48	29.79	127.2	-	135.11	-
Gly389	108.72	-	-	170.97	44.96	-	-	-	-	-
Ala390	121.81	-	-	174.54	49.54	18.01	-	-	-	-
Glu391	121.83	-	-	-	53.64	31.82	34.66	-	-	-

Table S3. Chemical-shift constrained (ϕ, ψ) torsion angles for the 24˚C P2R tau fibril core.

Residues	Phi (Φ)	Psi (Ψ)	Δ(Φ)	Δ (Ψ)	TALOS-N rank
Lys274					
Val275	-109.353	130.867	9.419	7.076	Strong
Gln276	-112.241	133.346	11.094	9.85	Strong
Ile277	-110.826	128.335	6.86	5.592	Strong
Ile278	-115.113	128.458	9.597	7.623	Strong
Asn279	-111.842	133.356	13.38	11.729	Strong
Lys280	-131.129	143.988	14.022	14.413	Strong
Lys281	-110.419	135.939	24.172	14.167	Generous
Leu282	-109.303	132.485	14.981	15.043	Strong
Asp283	-103.879	132.991	25.709	20.016	Strong
Leu284	-77.481	-26.889	14.654	20.901	Strong
Ser285	-148.275	157.569	12.876	10.296	Strong
Asn286	-119.358	138.618	20.312	13.072	Strong
Val287	-123.03	143.153	11.55	13.485	Strong
Gln288	-106.946	138.974	18.638	9.632	Generous
Ser289	-124.04	148.236	11.006	8.783	Strong
Lys290	-108.999	133.004	22.099	16.785	Warn
Cys291	-130.899	152.011	35.517	8.24	Strong
Gly292	-146.112	-176.472	57.171	30.181	Warn
Ser293	-103.132	155.686	20.707	13.552	Strong
Lys294	-106.478	136.969	25.009	12.727	Strong
Asp295	-130.988	149.07	12.98	12.821	Strong
Asn296	-126.762	143.525	12.195	11.119	Strong
Ile297	-115.475	135.002	16.641	12.494	Strong
Val300	-109.664	138.705	24.39	16.231	Strong
Pro301	-65.4	149.343	7.578	9.347	Strong
Gly302	154.878	-177.413	64.712	14.597	Warn
Gly303	-70.703	154.836	11.272	17.893	Warn
Gly304	133.446	-13.594	81.132	37.814	Warn
Gln307	-124.477	147.517	12.338	9.317	Strong
Ile308	-122.047	135.713	14.52	10.45	Strong
Val309	-105.868	127.732	9.89	7.241	Strong
Tyr310	-118.686	143.286	15.177	13.36	Strong
Lys311	-95.507	150.603	35.53	12.447	Strong
Pro312	-65.3	144.861	9.112	10.524	Strong
Val313	-113.586	135.098	16.278	9.78	Strong
Asp314	-107.215	131.59	16.176	9.719	Strong
Leu315	-114.67	132.53	19.981	15.46	Generous
Ser316	-135.456	158.635	16.07	12.626	Strong
Lys317	-128.886	142.896	12.225	9.19	Strong
Val318	-104.16	127.239	9.464	7.526	Strong
Thr319	-110.005	129.669	10.697	6.993	Strong
Ser320	-117.573	139.807	16.98	14.108	Strong
Lys370	-135.552	157.969	9.133	8.613	Strong
Ile371	-105.109	130.463	12.589	10.695	Strong

Table S4. Chemical-shift constrained (ϕ, ψ) torsion angles for the 12˚C P2R tau fibril core. The torsion angles for residues H268–G323 are used for simulated annealing analysis.

Residues	Phi (Φ)	Psi (Ψ)	Δ(Φ)	Δ (Ψ)	TALOS-N rank
His268	59.878	35.754	7.699	10.314	Strong
Gln276	-118.331	140.393	10.365	8.879	Strong
Ile277	-106.965	130.850	11.449	5.822	Strong
Ile278	-122.934	132.273	8.225	9.549	Strong
Asn279	-105.544	123.109	12.345	11.401	Strong
Lys280	-100.712	123.328	8.267	8.779	Strong
Lys281	-107.580	121.674	9.955	10.884	Strong
Leu282	-107.933	119.626	7.342	9.211	Strong
Asp283	-102.828	114.490	6.974	11.731	Strong
Leu284	-95.965	128.760	10.359	5.223	Strong
Ser285	-117.106	130.339	14.184	14.505	Strong
Asn286	-128.697	131.644	9.021	9.403	Strong
Val287	-119.075	133.734	12.278	8.749	Strong
Gln288	-132.299	150.248	7.342	9.880	Strong
Ser289	-123.311	143.842	11.692	20.318	Strong
Lys290	-69.226	-17.389	10.145	9.921	Strong
Cys291	-86.879	-5.327	12.885	9.756	Warn
Gly292	132.487	-174.329	40.722	19.168	Warn
Ser293	-128.375	142.090	21.218	9.722	Strong
Lys294	-130.712	142.941	13.807	13.855	Strong
Asp295	-93.005	135.368	28.323	12.484	Warn
Asn296	-128.800	148.920	12.789	11.815	Strong
Ile297	-120.942	131.016	13.637	9.715	Strong
Lys298	-106.256	121.782	17.820	11.544	Strong
His299	-114.355	125.420	11.488	7.747	Strong
Val300	-117.207	129.499	10.492	17.220	Strong
Pro301	-66.031	151.716	5.650	10.697	Strong
Gly302	-120.271	150.474	31.459	13.990	Warn
Gly303	-107.424	-172.838	24.485	21.174	Warn
Gly304	-175.192	165.681	63.105	56.035	Warn
Ser305	-149.329	159.574	9.461	7.186	Strong
Val306	-113.322	128.978	16.080	8.388	Strong
Gln307	-111.193	132.708	12.378	7.161	Strong
Ile308	-122.701	129.832	9.063	7.447	Strong
Val309	-107.895	129.058	10.254	7.264	Strong
Tyr310	-122.012	150.456	18.809	15.651	Strong
Lys311	-72.528	147.193	19.040	14.410	Strong
Pro312	-66.051	146.977	10.349	9.475	Strong
Val313	-109.382	131.553	15.178	11.303	Strong
Asp314	-96.548	118.665	11.157	11.380	Strong
Leu315	-86.627	118.630	15.833	10.311	Strong
Ser316	-96.887	-34.042	13.611	10.598	Generous
Lys317	-145.803	145.818	16.482	9.796	Strong
Val318	-129.221	126.158	8.056	6.893	Strong
Thr319	-104.814	127.966	11.663	7.801	Strong
Ser320	-118.366	141.874	16.967	21.808	Strong
Lys321	-75.998	-23.437	9.351	14.383	Strong
Cys322	-149.182	164.323	11.726	14.034	Strong
Gly323	-177.236	164.952	52.559	28.947	Warn

His329	-122.770	138.339	13.108	12.050	Strong
His330	-74.223	135.498	14.492	8.753	Strong
Lys331	-67.162	133.625	10.904	10.276	Strong
Gly355	-38.656	-28.168	77.291	30.296	Warn
Ser356	-149.597	151.449	9.998	8.788	Strong
Thr361	-101.896	128.347	10.130	7.725	Strong
His362	-121.573	148.672	15.753	12.700	Strong
Gly367	-61.602	-37.708	5.786	12.766	Warn
Asn368	-94.923	146.950	25.552	15.942	Warn
Glu372	-109.274	141.279	15.938	10.794	Strong
Thr373	-89.293	132.299	15.399	8.444	Strong
His374	-132.853	143.395	8.735	10.907	Strong
Lys375	-81.092	124.026	9.953	12.927	Strong
Ala382	-131.039	144.884	10.913	13.336	Strong
Lys383	-97.181	132.988	13.918	9.256	Strong
Ala384	-134.272	142.278	12.851	13.035	Strong
Lys385	-77.343	138.411	14.354	9.136	Generous
Thr386	-115.832	154.980	19.573	14.930	Warn
Asp387	-70.364	130.438	9.751	12.020	Strong
His388	-100.128	143.706	35.219	20.670	Warn
Gly389	87.855	-0.079	12.445	18.163	Warn
Ala390	-66.987	144.542	6.865	9.322	Strong
Glu391	-114.530	142.664	19.272	16.116	Strong

Table S5. β-strand positions in P2R tau fibrils, compared to the β-strands in full-length 0N4R tau (*18*). The strands are listed according to their approximate position alignment among the three samples.

**24˚C P2R fibrils**	**12˚C P2R fibrils**	**0N4R tau fibrils**
R2: ^{275}VQIINK^{280}	R2: ^{275}VQIIN KKLSN VQS^{283}	R2: ^{275}VQIIN^{279}
R2: ^{284}LSNVQSK^{290}		R2: ^{286}NVQS^{289}
R2: ^{294}KDNIK^{298}	R2: ^{295}NIKHVPG^{302}	R2: ^{293}SKDNI^{297}
R3: ^{306}VQIVYK^{311}	R3: ^{305}SVQIV YKPVD LSKV^{318}	R3: ^{306}VQIV^{309}
R3: ^{314}DLSKVTS^{320}		R3: ^{314}DLSKVTS^{320}
		R3: ^{336}QVEVK^{340}
	R': ^{381}NAKAKTD^{386}	

Table S6. Long-range (*i-j* ≥ 4) correlations of 24˚C P2R tau fibrils obtained from the 3D CCC DREAM-CORD spectrum. The first atom denotes the F1–F2 assignment while the second atom denotes the F3 assignment.

**Spectral assignment (F1-F2-F3)**	**Contacts ≤ 8.5 Å from XPLOR-NIH**
Ile277 Cβ – Ile277 Cδ1 – Ser289 Cα	Ser289 Cα
Pro312 Cα – Pro312 Cβ – Ser289 Cα	Ser289 Cα
Ile297Cβ – Ile297 Cγ – Ile308 Cα	Ile308 Cα
Val275 Cα – Val275 Cβ – Lys294 Cα or Ser289 Cα or Ser293 Cα	Ser293 Cα
Val275 Cα – Val275 Cβ – Cys291 Cβ or Lys317 Cγ	Cys291 Cβ
Val275 Cα – Val275 Cβ – Lys280 Cγ or Lys294 Cγ or Lys317 Cδ	Lys294 Cγ
Val275 Cβ – Val275 Cα – Cys291 Cβ or Lys317 Cγ	Cys291 Cβ
Val275 Cβ – Val275 Cα – Ile278 Cγ# or Lys311 Cγ or Ile308 Cγ#	Ile278 Cγ#
Val275 Cβ – Val275 Cα – Lys280 Cγ or Lys294 Cγ or Lys317 Cδ	Lys294 Cγ
Leu284 Cβ – Leu284 Cα – Cys291 Cβ or Lys317 Cγ	Lys317 Cγ
Leu284 Cα – Leu284 Cβ – Ser316 Cα or Lys281 Cα or Lys311 Cα	Ser316 Cα
Leu284 Cβ – Leu284 Cα – Val313 Cγ# or Val275 Cγ# or Val318 Cγ1	Val318 Cγ1
Ser285 Cα – Ser285 Cβ – Lys281 Cβ or Val313 Cβ or Val275 Cβ or Val318 Cβ	Val318 Cβ
Ser285 Cα – Ser285 Cβ – Ile308 Cβ or Asp314 Cβ or Gly304 Ca	Asp314 Cβ
Asn286 Cβ – Asn286 Cα – Lys281 Cγ or Leu315 Cδb	Leu315 Cδb
Val287 Cβ – Val287 Cα – Leu315 Cγ or Leu284 Cγ or Lys311 Cγ	Leu315 Cγ
Val287 Cβ – Val287 Cα – Asn279 Cb or Leu284 Cb or Ile297 Cb	Asn279 Cb
Val287 Cα – Val287 Cβ – Leu282 Cδ# or Leu284 Cδ#	Leu282 Cδ#
Gln288 Cβ – Gln288 Cα – Cys291 Cα or Ser285 Cα	Cys291 Cα
Gln288 Cα – Gln288 Cβ – Ile277 Cγ# or Ile297 Cγ# or Ile278 Cγ#	Ile277 Cγ#
Gln288 Cα – Gln288 Cβ – Cys291 Cα or Ser285 Cα	Cys291 Cα
Gln288 Cα – Gln288 Cβ – Leu315 Cβ or Lys317 Cε or Asn286 Cβ or Ile277 Cβ	Asn286 Cβ
Gln288 Cα – Gln288 Cβ – Lys290 Cγ or Ile308 Cγ# or Ile277 Cγ# or Pro301 Cγ or Ile278 Cγ#	Ile277 Cγ#
Ser289 Cβ – Ser289 Cα – Ile297 Cγ# or Ile277 Cγ#	Ile277 Cγ#
Ser289 Cβ – Ser289 Cα – Lys317 Cε or Asn286 Cβ or Ile277 Cβ	Ile277 Cβ
Ser289 Cα – Ser289 Cβ – Ile278 Cγ# or Ile297 Cγ# or Ile277 Cγ#	Ile277 Cγ#
Ser289 Cα – Ser289 Cβ – Val318 Cγ# or Val313 Cγ# or Val275 Cγ#	Val275 Cγ#
Ser289 Cα – Ser289 Cβ – Ile278 Cγ# or Ile308 Cγ#	Ile278 Cγ#
Ser289 Cα – Ser289 Cβ – Lys317 Cε or Asn286 Cβ or Ile277 Cβ or Leu315 Cβ	Ile277 Cβ
Ser289 Cα – Ser289 Cβ – Gln276 Cγ or Gln307 Cγ	Gln276 Cγ
Cys291 Cβ – Cys291 Cα – Leu282 Cγ or Pro312 Cγ	Pro312 Cγ
Cys291 Cβ – Cys291 Cα – Gln288 Cβ or Lys311 Cβ	Gln288 Cβ
Ser293 Cβ – Ser293 Cα – Val275 Cα or Val318 Cα or Thr319 Cα	Val275 Cα
Ser293 Cβ – Ser293 Cα – Ile308 Cγ# or Ile278 Cγ# or Pro301 Cγ or Ile277 Cγ#	Ile277 Cγ#
Val313 Cβ – Val313 Cα – Leu284 Cα or Gln288 Cα	Gln288 Cα
Val313 Cα – Val313 Cβ – Gln288 Cγ or Val287 Cβ	Gln288 Cγ

Table S7. Long-range (*i-j* ≥ 4) correlations of 12˚C P2R tau fibrils assigned from the 3D CCC CORD spectrum. Assignments are listed in the order of frequency dimensions F1–F2–F3. Bold entries are R2-R2 contacts that were used as dimer interface contacts in the structure calculation.

Long-range correlations (F1-F2-F3)	Contacts ≤ 8.5 Å from XPLOR-NIH
**Gln288 Cβ – Gln288 Cβ – Ile277 Cδ or Ile278 Cδ**	**Ile277 Cδ**
**Lys290 Cδ – Lys290 Cδ – Ile277 Cδ or Ile278 Cδ**	**Ile277 Cδ**
**Ile277 Cγ – Ile277 Cγ – Val313 Cγ or Val309 Cγ or Val287 Cγ**	**Val287 Cγ**
Ile297 Cδ – Ile297 Cδ – Val309 Cγ	Val309 Cγ
Ile297 Cβ – Ile297 Cβ – Val309 Cγ	Val309 Cγ
Ile297 Cγ – Ile297 Cγ – Val309 Cγ	Val309 Cγ
Val309 Cγ – Val309 Cγ – Ile297 Cβ	Ile297 Cβ
Ile297 Cδ – Ile297 Cδ – Ile308 Cα or Ile278 Cα	Ile308 Cα
Ile297 Cδ – Ile297 Cδ – Tyr310 Cβ or Lys321 Cε or Asp283 Cβ	Tyr310 Cβ
Ile297 Cδ – Ile297 Cδ – IIle308 Cγ or Ile278 Cγ	Ile308 Cγ
Val313 Cγ or Val309 Cγ – Val313 Cγ or Val309 Cγ – Val318 Cγ or Ile297 Cβ	Ile297 Cβ
Ile297 Cγ or Cys291 Cβ – Ile297 Cγ or Cys291 Cβ – Lys267 Cδ or Val309 Cγ	Val309 Cγ
Ile297 Cδ – Ile297 Cγ or Cys291 Cβ – Val313 Cγ or Val309 Cγ ορ Val287 Cγ	Val309 Cγ
Ile308 Cδ – Ile308 Cδ – Ile297 Cγ or Lys267 Cδ or Leu315 Cγ	Ile297 Cγ
Ile308 Cδ – Ile308 Cδ – Asp314 Cβ or Ile278 Cβ or Ile297 Cβ	Ile297 Cβ
Ser316 Cβ – Ser316 Cβ – L284 Cγ or L282 Cγ	L284 Cγ
Ser316 Cα – Ser316 Cα – Tyr310 Cβ or Asp283 Cβ or Lys321 Cε	Asp283 Cβ
Ser316 Cβ – Ser316 Cβ – Asp283 Cα or His299 Cα or Gln288 Cα	Asp283 Cα
Ser316 Cβ – Ser316 Cβ – Ser285 Cβ or Ser320 Cβ	Ser285 Cβ

Table S8. XPLOR-NIH parameters for calculating the P2R tau fibril core structures.

	24˚C P2R tau fibrils			
**XPLOR-NIH Potential**	**Experimental Basis**	**Restraints per monomer 274-321**	**Round 1 Scale Factor**	**Best model energy (kcal/mol)**
PosDiffPot (ncs)	^{13}C and ^{15}N chemical shifts	-	1000	2.56
RDCPot	cross-β hydrogen bonds	-	1-30	0.83
CDIH (dihedral angles)	TALOS-N predictions based on chemical shifts	42 in (274-321) construct	10-200	26.59
NOE (long-range contacts)	Long-range cross peaks in CCC spectrum	33+3 nmono	0.1-30	34.25
NOE (medium-range contacts)	Medium-range cross peaks in CCC spectrum	103	0.1-30	34.25
ResAff	Standard	-	1	-3.07
HBDB	Standard	-	1	-413.3
VDW	Standard	-	0.004-4	288.49
TorsionDB	Database	-	0.0001-0.05	945.29
BOND	Standard bond lengths	-	1	126.01
ANGL	Standard bond angles	-	0.4-1	403.4
IMPR	Standard bond geometry	-	0.1-1	50.13
	12˚C P2R tau fibrils			
**XPLOR-NIH Potential**	**Experimental Basis**	**Restraints per monomer 264-326**	**Round 1 Scale Factor**	**Best model energy (kcal/mol)**
PosDiffPot (ncs)	^{13}C and ^{15}N chemical shifts	-	1000	5.65
RDCPot	cross-β hydrogen bonds	-	1-10	1.06
CDIH (dihedral angles)	TALOS-N predictions based on chemical shifts	49 in (264-326) construct	10-200	48.89
NOE (long-range contacts)	Long-range cross peaks in CCC spectrum	16 nmono and 3 explicit inter	0.5-40 (inter) 0.1-40 (intra)	9.85+0.14
ResAff	Standard	-	1	1.56
HBDB	Standard	-	1	-1097.28
VDW	Standard	-	0.004-4	598.77
TorsionDB	low energy sidechain conformations	-	0.0001-0.05	603.16
BOND	Standard bond lengths	-	1	184.48
ANGL	Standard bond angles	-	0.4-1	654.89
IMPR	Standard bond geometry	-	0.1-1	57.87

Table S9. Solid-state NMR experimental parameters for P2R tau fibrils. All experiments were recorded on an 18.8 T spectrometer (800 MHz ^{1}H frequency). Reported temperatures are estimated sample temperatures based on the water ^{1}H chemical shift.

Experiment	NMR Parameters	Expt. Time
24˚C P2R tau fibrils		
2D CC with 23 ms CORD mixing	T_{sample} = 278 K; ν_{MAS} = 10.5 kHz, ns = 32, τ_{rd} = 1.5 s, $t_{1,max}$ = 9.9 ms; $t_{1,inc}$ = 24.8 μs; τ_{dwell} = 6 μs; τ_{acq} = 15.4 ms; τ_{HC} = 70 μs; τ_{CORD} = 23 ms; ν_{1Hacq} = 71 kHz TPPM	11 hr
2D NCA SPECCP	T_{sample} = 278 K; ν_{MAS} = 10.5 kHz, ns = 144, τ_{rd} = 1.7 s, $t_{1,max}$ = 14.3 ms; $t_{1,inc}$ = 95.2 μs; τ_{dwell} = 6 μs; τ_{acq} = 15.4 ms; τ_{HN} = 1 ms; τ_{NC} = 5.5 ms; $\nu_{15NspecificCP}$ = 26.3 kHz ramp 90-100%; $\nu_{13CspecificCP}$ = 15.8 kHz; $\nu_{1HspecificCP}$ = 71 kHz CW; ν_{1Hacq} = 71 kHz TPPM	21 hr
3D hNCACX SPECCP	T_{sample} = 281 K; ν_{MAS} = 14 kHz, ns = 32, τ_{rd} = 1.7 s, $t_{1,max}$ = 8.6 ms; $t_{1,inc}$ = 142.8 μs; $t_{2,max}$ = 6.4 ms; $t_{2,inc}$ = 142.9 μs; τ_{dwell} = 6 μs; τ_{acq} = 15.4 ms; τ_{HN} = 1 ms; τ_{NC} = 4.5 ms; $\nu_{1HspecificCP}$ = 83 kHz CW; ν_{1Hacq} = 83 kHz TPPM, τ_{CORD} = 82 ms	176 hr
3D hNCOCX SPECCP	T_{sample} = 281 K; ν_{MAS} = 14 kHz, ns = 64, τ_{rd} = 1.7 s, $t_{1,max}$ = 7.1 ms; $t_{1,inc}$ = 142.8 μs; $t_{2,max}$ = 6 ms; $t_{2,inc}$ = 142.9 μs; τ_{dwell} = 6 μs; τ_{acq} = 15.4 ms; τ_{HN} = 1 ms; τ_{NC} = 4.5 ms; $\nu_{1HspecificCP}$ = 83 kHz CW; ν_{1Hacq} = 83 kHz TPPM, τ_{CORD} = 82 ms	184 hr
3D CONCA SPECCP	T_{sample} = 281 K; ν_{MAS} = 14 kHz, ns = 16, τ_{rd} = 1.7 s, $t_{1,max}$ = 6.4 ms; $t_{1,inc}$ = 214.3 μs; $t_{2,max}$ = 7.8 ms; $t_{2,inc}$ = 142.9 μs; τ_{dwell} = 6 μs; τ_{acq} = 15.4 ms; τ_{HC} = 1.1 ms; τ_{CN} = 4.5 ms; τ_{NC} = 4.5 ms; $\nu_{1HspecificCP}$ = 83 kHz CW; ν_{1Hacq} = 71 kHz TPPM	51 hr
1D ^{13}C DP	T_{sample} = 278 K; ν_{MAS} = 10.5 kHz, ns = 1024, τ_{rd} = 3 s, τ_{dwel} = 6 μs; τ_{acq} = 15.4 ms; ν_{1Hacq} = 83 kHz TPPM	0.85 hr
1D ^{13}C CP	T_{sample} = 278 K; ν_{MAS} = 10.5 kHz, ns = 1024, τ_{rd} = 1.7 s, τ_{dwel} = 6 μs; τ_{acq} = 15.3 ms; τ_{HC} = 70 μs; ν_{1Hacq} = 71 kHz TPPM	0.5 hr
2D water-edited NCA - 4 ms	T_{sample} = 278 K; ν_{MAS} = 10.5 kHz, ns = 280, τ_{rd} = 1.7 s; $t_{1,max}$ = 7.6 ms; $t_{1,inc}$ = 95.2 μs; τ_{dwell} = 6 μs; τ_{acq} = 12.3 ms $\tau_{1Hselpulse}$ = 950 μs; $\tau_{T2filter}$ = 95.2 μs; τ_{SD} = 4 ms; τ_{HN} = 1 ms; τ_{NC} = 4 ms; $\nu_{1HspecificCP}$ = 71 kHz CW; ν_{1Hacq} = 71 kHz TPPM;	22 hr
2D water-edited NCA - 100 ms	T_{sample} = 278 K; ν_{MAS} = 10.5 kHz, ns = 144, τ_{rd} = 1.7 s; $t_{1,max}$ = 7.6 ms; $t_{1,inc}$ = 95.2 μs; τ_{dwell} = 6 μs; τ_{acq} = 12.3 ms $\tau_{1Hselpulse}$ = 950 μs; $\tau_{T2filter}$ = 95.2 μs; τ_{SD} = 100 ms; τ_{HN} = 1 ms; τ_{NC} = 4 ms; $\nu_{1HspecificCP}$ = 71 kHz CW; ν_{1Hacq} = 71 kHz TPPM;	12 hr
3D CCC: 2.5 ms DREAM – 400 ms CORD	T_{sample} = 281 K; ν_{MAS} = 14 kHz, ns = 12, τ_{rd} = 1.6 s, $t_{1,max}$ = 5.1ms; $t_{1,inc}$ = 57.2 μs; $t_{2,max}$ = 5.1 ms; $t_{2,inc}$ = 57.2 μs; τ_{dwell} = 6 μs; τ_{acq} = 15.6 ms; τ_{HC} = 170 μs; τ_{CORD} = 400 ms; τ_{DREAM} = 2.5 ms; ν_{1Hacq} = 71 kHz TPPM, $\nu_{1Hdream}$ = 83 kHz CW	219 hr
12˚C P2R tau fibrils		
2D CC with 23 ms CORD mixing	T_{sample} = 278 K; ν_{MAS} = 10.5 kHz, ns = 64, τ_{rd} = 1.6 s, $t_{1,max}$ = 7.5 ms; $t_{1,inc}$ = 24.8 μs; τ_{dwell} = 6 μs; τ_{acq} = 15.4 ms; τ_{HC} = 70 μs; τ_{CORD} = 23 ms; ν_{1Hacq} = 83 kHz TPPM	18 hr
2D NCA SPECCP	T_{sample} = 278 K; ν_{MAS} = 10.5 kHz, ns = 136, τ_{rd} = 1.7 s, $t_{1,max}$ = 14.3 ms; $t_{1,inc}$ = 95.2 μs; τ_{dwell} = 6 μs; τ_{acq} = 18.4 ms; τ_{HN} = 1 ms; τ_{NC} = 5 ms; $\nu_{15NspecificCP}$ = 26.3 kHz ramp 90-100%; $\nu_{13CspecificCP}$ = 15.8 kHz; $\nu_{1HspecificCP}$ = 83 kHz CW; ν_{1Hacq} = 71 kHz TPPM	23 hr
3D hNCACX SPECCP	T_{sample} = 278 K; ν_{MAS} = 14 kHz, ns = 40, τ_{rd} = 1.7 s, $t_{1,max}$ = 6.8 ms; $t_{1,inc}$ = 142.8 μs; $t_{2,max}$ = 4.8 ms; $t_{2,inc}$ = 142.9 μs; τ_{dwell} = 6 μs; τ_{acq} = 15.4 ms; τ_{HN} = 1 ms; τ_{NC} = 5 ms; $\nu_{1HspecificCP}$ = 83 kHz CW; ν_{1Hacq} = 83 kHz TPPM, τ_{CORD} = 82 ms	131 hr

3D hNCOCX SPECCP	T_{sample} = 278 K; ν_{MAS} = 14 kHz, ns = 32, τ_{rd} = 1.7 s, $t_{1,max}$ = 6.8 ms; $t_{1,inc}$ = 142.8 µs; $t_{2,max}$ = 4.5 ms; $t_{2,inc}$ = 142.9 µs; τ_{dwell} = 6 µs; τ_{acq} = 15.4 ms; τ_{HN} = 1 ms; τ_{NC} = 4.5 ms; $\nu_{1HspecificCP}$ = 83 kHz CW; ν_{1Hacq} = 83 kHz TPPM, τ_{CORD} = 82 ms	52 hr
3D CONCA SPECCP	T_{sample} = 281 K; ν_{MAS} = 14 kHz, ns = 24, τ_{rd} = 1.7 s, $t_{1,max}$ = 4.9 ms; $t_{1,inc}$ = 214.3 µs; $t_{2,max}$ = 6.8 ms; $t_{2,inc}$ = 142.9 µs; τ_{dwell} = 6 µs; τ_{acq} = 15.4 ms; τ_{HC} = 1 ms; τ_{CN} = 4.5 ms; τ_{NC} = 5 ms; $\nu_{1HspecificCP}$ = 83 kHz CW; ν_{1Hacq} = 83 kHz TPPM	52 hr
3D CCC:<br>24 ms CORD –<br>404 ms CORD	T_{sample} = 281 K; ν_{MAS} = 14 kHz, ns = 16, τ_{rd} = 1.5 s, $t_{1,max}$ = 4.28 ms; $t_{1,inc}$ = 57.1 µs; $t_{2,max}$ = 4.11ms; $t_{2,inc}$ = 57.1 µs; τ_{dwell} = 6 µs; τ_{acq} = 15.6 ms; τ_{HC} = 500 µs; τ_{CORD2} = 404 ms; τ_{CORD1} = 24 ms; ν_{1Hacq} = 71 kHz TPPM	188 hr
1D ^{13}C DP	T_{sample} = 278 K; ν_{MAS} = 10.5 kHz, ns = 16, τ_{rd} = 3 s, τ_{dwel} = 6 µs; τ_{acq} = 15.4 ms; ν_{1Hacq} = 83 kHz TPPM	60 sec
1D ^{13}C CP	T_{sample} = 278 K; ν_{MAS} = 10.5 kHz, ns = 16, τ_{rd} = 1.7 s, τ_{dwel} = 6 µs; τ_{acq} = 15.3 ms; τ_{HC} = 70 µs; ν_{1Hacq} = 71 kHz TPPM	30 sec

Symbols: T_{sample} = sample temperature; ν_{MAS} = MAS frequency; ns = number of scans (transients) per free induction decay (FID); τ_{rd} = recycle delay between scans; $t_{1,max}$ = maximum t_1 (indirect dimension 1) evolution time; $t_{1,inc}$ = increment for t_1 (indirect dimension 1) evolution time; τ_{dwell} = dwell time during direct FID acquisition; τ_{acq} = maximum acquisition time during direct FID detection; τ_{XY} = cross polarization (CP, ^{SPECIFIC}CP, BSH-CP, or DREAM) contact time during CP from channel X to channel Y; $\nu_{nuc\text{-}CP,XY}$ = radiofrequency field strength for CP spin lock on *nuc* (*nuc* = ^{1}H, ^{13}C, ^{15}N) during CP from X to Y; $\nu_{1H,\,acq}$ = dipolar decoupling field strength during FID acquisition and indirect dimension evolution; $\nu_{nuc\text{-}DCP}$ = rf spin lock field strength on *nuc* during heteronuclear CP; $\nu_{1H\text{-}DD,DCP}$ = ^{1}H dipolar decoupling field strength during heteronuclear CP; τ_{DREAM} = spin lock contact time during homonuclear coherence transfer with the DREAM condition.
